# The Association Between Health‐Related Quality of Life Scores and Clinical Outcomes for People Living With Lung Cancer: An Australian Registry Cohort Study Using Patient‐Reported Outcomes to Drive Value‐Based Healthcare

**DOI:** 10.1111/1759-7714.70245

**Published:** 2026-02-23

**Authors:** Susan V. Harden, Madeleine T. King, Jing Jing Li, Sanuki Tissera, Mike Lloyd, Lisa Briggs, Tom Wood, Baki Billah, Dani Samankula, Shantelle Smith, Margaret Brand, Tali Lang, Philip Parente, Sarah McGrath, David Langton, Tegan Dumnall, Barton Jennings, Sandra Nicholls, Rob G. Stirling, Gary Richardson, John Zalcberg

**Affiliations:** ^1^ School of Public Health and Preventive Medicine Monash University Melbourne Australia; ^2^ Department of Radiation Oncology Peter MacCallum Cancer Centre Melbourne Australia; ^3^ Sir Peter MacCallum Dept of Oncology University of Melbourne Melbourne Australia; ^4^ School of Psychology University of Sydney Sydney Australia; ^5^ Centre for Health Economics Monash University Melbourne Australia; ^6^ Cabrini Hospital Melbourne Australia; ^7^ Central Clinical School, Department of Medicine, Nursing and Health Sciences Monash University Melbourne Australia; ^8^ Department of Oncology Eastern Health Melbourne Australia; ^9^ Department of Thoracic Medicine Peninsula Health Melbourne Australia; ^10^ Department of Respiratory Medicine Monash Health Melbourne Australia; ^11^ Department of Respiratory Medicine Alfred Health Melbourne Australia; ^12^ Department of Medical Oncology Alfred Health Melbourne Australia

**Keywords:** guideline concordant treatment, health‐related quality of life, lung cancer, patient reported experience measures, patient reported outcome measures, value based healthcare

## Abstract

**Introduction:**

Improving patient‐centered outcomes is a core aim of value‐based healthcare (VBHC). Integrating patient‐reported outcome and experience measures (PROMs/PREMs) into clinical quality registries may provide insight into health‐related quality of life (HRQL) and variation in care. We piloted PROMs/PREMs collection in an Australian Lung Cancer Registry to evaluate associations between HRQL, clinical outcomes and treatment value.

**Methods:**

Individuals newly diagnosed with lung cancer across five metropolitan health services were invited to complete electronic PROMs (EORTC QLQ‐C30 and QLQ‐LC29) and PREMs at baseline and follow‐up. Preference‐based utilities (QLU‐C10D) and quality‐adjusted life‐years (QALYs) were derived and linked with registry clinical data. Stage‐specific Australian health system cost estimates for guideline concordant treatment (GCT) provided context for value‐based reporting. Multivariable regression examined associations between HRQL and clinical variables.

**Results:**

Baseline PROMs/PREMs were completed by 241/490 (49%) participants. HRQL was associated with cancer stage, ECOG performance status ≥ 2, comorbidities, weight loss, and receipt of GCT (*p* = 0.041). HRQL remained stable among ongoing respondents over time. Estimated health system costs increased with advancing stage, while earlier stage disease was associated with better HRQL and survival. A registry‐level VBHC dashboard integrating HRQL, patient experience, clinical quality indicators and cost context was developed to support health service performance review.

**Conclusions:**

PROMs/PREMs linked with clinical and cost data provided meaningful insight into patient‐centered outcomes and drivers of value in lung cancer care. This VBHC framework highlights the importance of early diagnosis and access to evidence‐based treatment and offers a scalable approach to support patient‐centered quality improvement at the health system level.

## Graphical abstract

Linking patient‐reported outcomes and experiences with clinical and cost data reveals key drivers of value in lung cancer care, empowering earlier diagnosis, equitable access, and patient‐centered quality improvement.

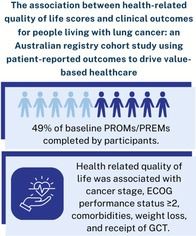

## 1 Introduction

There have been multiple paradigm shifts in the treatment of lung cancer in recent years following the publication of practice‐changing trials demonstrating improved overall survival for all stages of disease [1, 2, 3, 4, 5, 6, 7, 8, 9]. People diagnosed with lung cancer are living longer in this new era of personalized medicine with the addition of immunotherapy and oncogene‐targeted treatments combined with advances in surgery, radiotherapy and chemotherapy. This substantial increase in life span has empowered the patient voice for lung cancer, supported by increasing numbers of global patient advocacy organizations contributing to healthcare policy and decision making. Health system expenditure on lung cancer is also escalating while financial resources remain finite for all governments [10, 11]. An Italian study reported a significant increase in average overall patient costs following the introduction of immunotherapy for advanced lung cancers as well as a significant reduction in mortality [12].

The concept of improving value‐based healthcare (VBHC) was first introduced in the United States in 2006 [13, 14]. The goal of VBHC is ultimately to improve health outcomes that are important to people, relative to the cost of that care [15, 16]. A value‐based framework for reimbursing lung cancer care was considered in the US, where the main focus was at the health service provider level to reduce costs incurred for low‐value interventions, such as PET imaging for metastatic disease and intense radiologic follow‐up after curative‐intent therapy [17]. However, from a patient‐centered perspective, this issue is more complex. For example, follow‐up scanning may provide valued reassurance to patients, despite associated potential out‐of‐pocket and health system costs.

Patient‐centered aspects of care, such as shared decision‐making, information, education, and wellbeing, are of enormous importance to the community generally and should be considered alongside overall survival and costs across all stages of lung cancer [18]. Therefore, to make informed decisions about how to provide VBHC, patient‐centered health priorities must be identified, defined, and measured before and after the addition of new interventions. This approach could reveal a comprehensive picture of added value from the patient perspective, as well as from medical and financial perspectives.

Lung cancer registries have lagged behind other population cancer registries in routinely collecting patient‐reported measures [19, 20] While patient‐reported outcome measures (PROMs) are well‐established in clinical trials of lung cancer treatment [21], PROMs and patient‐reported experience measures (PREMs) collection in real‐world lung cancer settings has emerged only recently [22, 23, 24, 25]. The Danish lung cancer registry reported a 58% response rate to PROMs when collected locally during attendance at participating hospitals [22].

The Victorian Lung Cancer Registry (VLCR) collects population‐based clinical data [26], reporting a series of clinical quality indicators based on the Australian lung cancer Optimal Care Pathway guidelines and international guidelines for best practice [27, 28, 29, 30, 31]. Optimal guideline concordant treatment (GCT) was associated with improved survival outcomes in an American real world database [32]. We have also shown this to be the case within our Australian registry population for NSCLC and SCLC [33, 34] and have proposed using GCT as a novel clinical quality indicator for cancer registries measuring the impact of evidence‐based practice on real world outcomes.

We hypothesized that introducing PROMs/PREMS collection to our registry might enable the registry to explore how health‐related quality of life (HRQL) and patient satisfaction were associated with cancer stage and optimal treatment. Furthermore, we hypothesized that by associating HRQL with current estimates of Australian costs for stage‐specific optimal lung cancer treatments, the registry could create a value‐based framework for patient‐centered quality improvement initiatives during this time of rapidly evolving increased use of high‐cost systemic treatment options, increasing survival, rising health costs and greater inclusion of the consumer voice [35]. We aimed to better understand the role PROMs and PREMs could play in VBHC and to explore whether a value‐based framework for improving health outcomes could be developed for the VCLR by assessing health‐related quality of life (HRQL), clinical data, and costs for health outcomes that matter to patients.

To our knowledge, this pilot study is the first to integrate PROMs/PREMs with clinical treatment and health system costs for optimal treatments within a lung cancer registry, demonstrating how registry‐level patient‐reported data might inform value‐based healthcare.

Primary objectives of our pilot study were (1) measuring response rates to serial PROMs/PREMs surveys, (2) reporting of the PROMs/PREMs responses, (3) calculation of preference‐based HRQL, and correlation of HRQL and PROMs domain scores, (4) deriving contemporary estimates of Australian costs for optimal lung cancer treatment, and (5) creating a template value‐based registry dashboard combining PROMs/PREMs and costs with routinely collected clinical outcome data.

Secondary objectives were correlation of patient reported HRQL and overall satisfaction with routinely collected clinical variables, exploratory reporting of our novel co‐designed PREMs questions and assessing the utility and scalability of PROMS/PREMs collection and value‐based dashboard reporting across the whole lung cancer registry.

## 2 Methods

### 2.1 Study Design and Participants

This was a prospective, proof‐of‐principle feasibility cohort study, conducted from April 2021 to April 2022 to collect PROMs/PREMs to inform VBHC from at least 200 people newly diagnosed with lung cancer across five pilot metropolitan health services (four public, one private), within the VLCR [26]. The VLCR captures diagnostic, management and clinical outcomes data from patients diagnosed within 19 health care networks (50 hospitals) across the State of Victoria, Australia using an opt‐out consent model where patients are notified that they will be entered into the registry unless they request not to be. Data are collected and collated through direct downloads from hospital information systems, supplemented by individual abstraction of clinical records [36].

After the 2 weeks opt‐out period, VLCR participants from the 5 pilot health services were invited to complete a series of PROMs/PREMs electronic surveys sent via SMS or email. Respondents to this survey invitation were defined as the VBHC study cohort. For this pilot, due to funding and ethical limitations, we were unable to contact non‐responders to ascertain the reasons for their non‐response. We estimated a response rate of around 40%–50%, based on the Danish lung cancer registry PROMs data and factoring in a lower response rate for centrally sent electronic surveys and took the pragmatic decision to continue inviting new registry participants to complete PROMs/PREMs until at least 200 PROMs/PREMs responses had been received.

### 2.2 Data Collection

#### 2.2.1 Clinical Variables

Patient and disease characteristics, diagnostic procedures/tests, treatments (systemic anti‐cancer therapy (SACT), radiotherapy and surgery), and overall survival were routinely collected. Performance status was recorded using the Eastern Conference Oncology Group‐ Performance Status (ECOG‐PS) categories [37]. Smoking status was categorized as current, former or never. Socioeconomic status was ranked based on population‐based quintiles using the Index of Relative Socio‐economic Disadvantage [38]. The notifying hospital was classified as public or private.

Clinical stage at diagnosis was classified according to the clinical stage documented in the medical records, based on the 8th edition of the IASLC TNM Classification for Lung Cancer [39]. Non‐small cell lung cancer (NSCLC) and small cell lung cancer (SCLC) were based on the ICD‐10 morphology classification [40]. Definitions of comorbidities have been reported [41]. Weight loss was dichotomously recorded as no weight loss, weight loss, or not documented.

Guideline Concordant Treatments (GCT) for NSCLC were defined as follows: stage I and stage II—surgery or radiotherapy; stage III—SACT with either surgery or radiotherapy; stage IV—SACT alone. For SCLC, GCT definitions were as follows: limited stage I–III—SACT with either radiotherapy or surgery; extensive stage IV—SACT alone [32, 33, 34].

Overall survival (OS) for patients was calculated using the time between date of diagnosis and date of death or (censored date 05/05/2023) using data obtained from the Victorian Registry of Births, Deaths and Marriages.

#### 2.2.2 PROMs Survey for HRQL

HRQL was assessed with two validated PROMs from the European Organization for Research and Treatment of Cancer (EORTC) Quality of Life Questionnaire (QLQ) suite: the 30‐item core module (QLQ‐C30) [42] and the 29‐item lung cancer module (QLQ‐LC29) [43]. PROMs were completed at up to four timepoints. HRQL outcome scores were derived from the data collected using these two questionnaires, following the EORTC standard QLQ scoring procedures [44]. Preference‐based health utility scores (QLU‐C10D) were calculated from the QLQ‐C30 data using the Australian EORTC QLU‐C10D utility algorithm [45, 46]. Serial QLU‐C10D scores were then used to calculate quality‐adjusted life years (QALYs). Further details on questionnaire content and scoring are provided as a supplementary PROMs/PREMs appendix: Tables S3 and S6.

#### 2.2.3 PREMs Survey

Two customized lung cancer‐specific experience surveys were co‐designed with details on content and scoring provided in the PROMs/PREMs appendix Table S4 (Baseline) and Table S5 (Final—9 months later). The surveys used 23 validated questions with positive response scoring from the Victorian Agency for Health Information 2019 Patient Experience of Cancer Care Survey project, with overall satisfaction with care used for the primary PREMs outcome measure (www.health.vic.gov.au/health‐strategies/victorian‐cancer‐patient‐experience‐survey‐tool‐project). The co‐designed surveys also included content‐validated questions, customized for a lung cancer cohort based on extensive consumer input (authors L.B., T.W.), the Lung Foundation Australia consumer forum and our previously reported qualitative study of 14 semi‐structured consumer interviews regarding PROMs/PREMs [47]. These exploratory PREMs questions were selected to cover five novel patient‐identified themes important for wellbeing but lacking from existing validated PROMs, including personal attitude to their lung cancer diagnosis, maintaining independence, support from their relationships with family, friends, and their medical team, and addressing lack of awareness of lung cancer [47]. Questions on shared decision making, confidence in the treating team, receiving best available care, and access to cancer nurse specialists for support were viewed by our consumer groups as especially important. Reliability and face validity of responses were assessed during the pilot study.

#### 2.2.4 PROMs/PREMs Scheduling

A secure electronic system, eCaptis (www.Medicity.com.au), was used for scheduling and collecting PROMs/PREMs data. Four PROMs surveys were scheduled: baseline (around the time of diagnosis), 3 months (at the conclusion of first‐line treatment), 6 months, and 9 months. The QLQ‐C30 was scheduled for all four timepoints, while the QLQ‐LC29 was scheduled for 3 and 6 months in consideration of patient burden, with PREMs surveys scheduled at baseline and 9 months. An SMS reminder was sent for each survey instrument after 2 weeks if not returned. Subsequent surveys were only sent to participants who had responded to a prior survey, following a check of their ongoing health status.

#### 2.2.5 Health System Costs

Stage‐specific health system cost estimates were included to contextualize HRQL findings within a value‐based care framework. We used lung cancer treatment costs from available but historic published Australian data [11] and updated stage specific costs during the first year of treatment in accordance with a more contemporary report on cost‐effectiveness of lung cancer screening in Australia [48], and a recently published European study incorporating modern optimal treatment costs since the introduction of immunotherapies and oncogene‐driven targeted treatments [12]. All costs were reported in Australian dollars (AUD 2021). Cost ratios by stage from the European study were utilized to estimate separate Australian costs for stages I and II treatments in the primary analysis. A sensitivity analysis was used to estimate costs for stages III and IV relative to stage I as a robustness check. Although Buja et al. reported costs for the European context, the treatments available were consistent with those available in Australia, providing an up to date validity check given that the data reported in Goldsbury et al. only included costs up to 2013 [11].

### 2.3 Statistical Analyses

Percentages were used to summarize categorical data. Chi‐square test or Fisher Exact Test was used to evaluate the association between two categorical variables. Pearson's correlation coefficient was used to assess correlation between the EORTC QLU‐C10D health utility score and the various EORTC QLQ‐C30 and QLQ‐LC29 domain scores. A Kruskal‐Wallis test was used to determine if there were significant differences between the serial QLU‐C10D surveys. The Kaplan–Meier and Cox Proportional Hazard estimates of survival and the log‐rank test were used for comparing survival between groups (e.g., cancer stages). Two separate multivariable linear regression analyses were completed to identify factors correlated with QLU‐C10D scores and QALYs. A stepwise variable selection method based on minimizing Akaike Information Criterion (AIC) score was used to select variables to use in the multivariable models. A *p*‐value of 0.05 or less was considered statistically significant for the multivariable analyses. A post hoc analysis comparing the VBHC cohort to the overall VLCR cohort was performed to assess study selection and non‐response bias. Statistical analyses were performed using The R Project for Statistical Computing (https://www.r‐project.org).

### 2.4 Ethics Statement

The VLCR has National Mutual Acceptance (NMA) ethics approval to collect data for patients diagnosed with primary lung cancer using an opt‐out consent model [HREC/16/Alfred/84]. An ethics application for the VBHC sub‐study was approved by Monash University HREC [Project 26 768, 20/10/2020]. Governance approval to invite eligible patients to the VBHC study was obtained from each participating health service.

## 3 Results

### 3.1 VBHC Cohort

**FIGURE 1:**
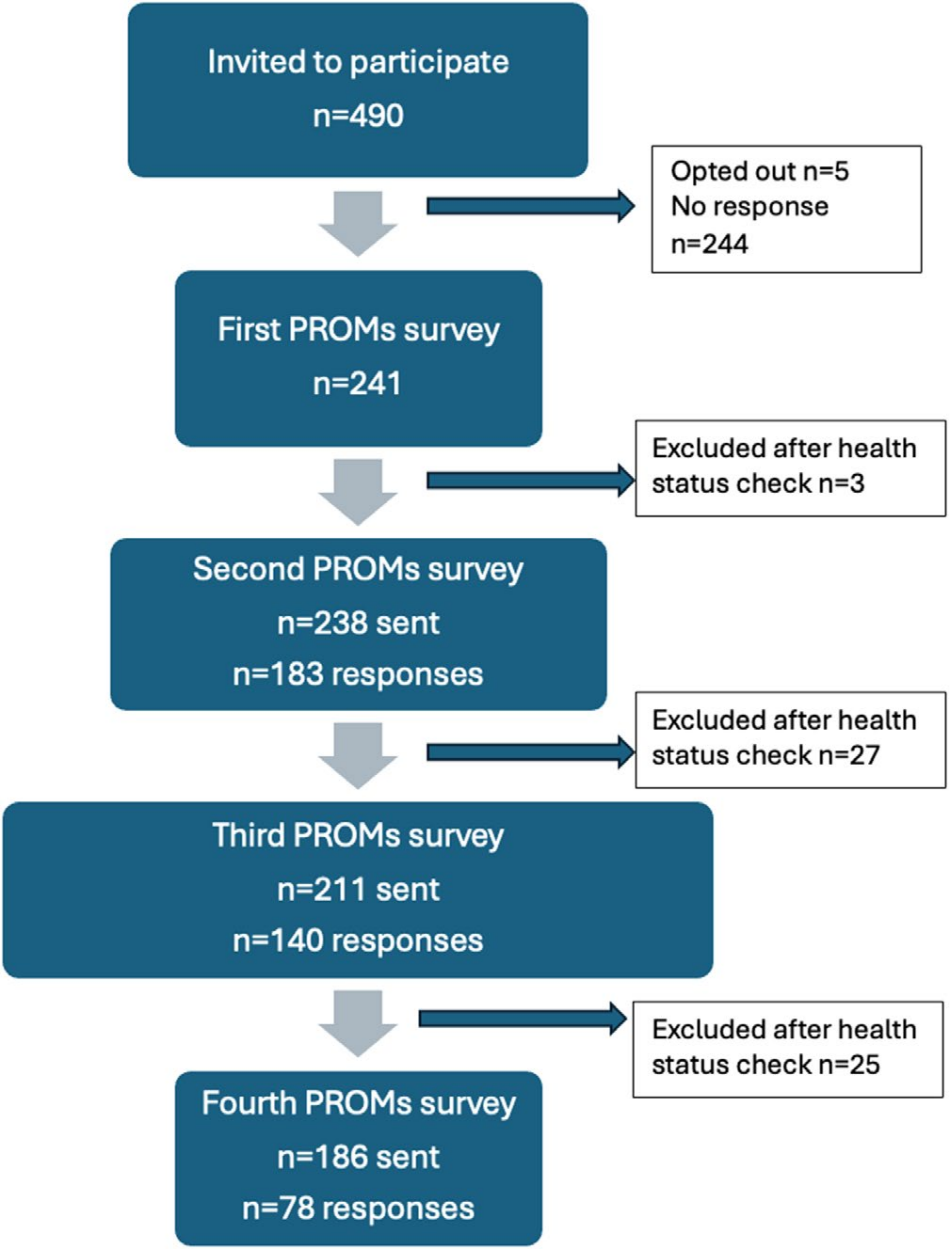
STROBE recruitment flow diagram for lung cancer registry value‐based healthcare pilot study.

In total 241/490 (49%) people diagnosed with lung cancer at 5 participating pilot VLCR health services and invited to be part of the study completed the first PROMs/PREMs survey and were included in the pilot value‐based healthcare cohort analysis, Figure 1.

### 3.2 Clinical Characteristics

Clinical and demographic characteristics of the VBHC cohort are shown in Table 1. Histologic diagnosis was NSCLC in 229 (95%) participants and SCLC in 12 (5%). Cancer staging at diagnosis was documented in 209/241 (87%) cases. ECOG performance status was documented in 180/241 cases (75%). Smoking status was documented in 221/241 cases (92%). Case discussion at a Multi‐disciplinary meeting (MDM) took place for 157 (87%) participants, with no record of this in 24 (13%).

**TABLE 1:** Baseline characteristics of the cohort that opted into the study.

Patient Characteristic	All patients *n* = 241 [*n*(%)]	GCT *n* = 181 [*n*(%)]	Non‐GCT *n* = 28 [*n*(%)]	*p*
Age				0.004
< 60	40 (16.6)	33 (18.2)	1 (3.6)	
60–69	71 (29.5)	49 (27.1)	12 (42.9)	
70–79	101 (41.9)	82 (45.3)	7 (25.0)	
80 years and over	29 (12.0)	17 (9.4)	8 (28.6)	
Gender				0.7
Female	120 (49.8)	90 (49.7)	15 (53.6)	
Male	121 (50.2)	91 (50.3)	13 (46.4)	
Cancer type				> 0.9
NSCLC^a^	229 (95.0)	175 (96.7)	27 (96.4)	
SCLC^b^	12 (5.0)	6 (3.3)	1 (3.6)	
Stage at diagnosis				< 0.001
Stage I	75 (35.9)	74 (40.9)	1 (3.6)	
Stage II	31 (14.8)	30 (16.6)	1 (3.6)	
Stage III	38 (18.2)	18 (9.9)	20 (71.4)	
Stage IV	65 (31.1)	59 (32.6)	6 (21.4)	
Stage Unknown	32	—	—	
ECOG^c^ status				< 0.006
0–1	160 (88.9)	124 (93.2)	19 (76.0)	
≥ 2	20 (11.1)	9 (6.8)	6 (24.0)	
Unknown	61	—	—	
Smoking status				0.8
Never smoked	41 (18.6)	33 (19.8)	4 (15.4)	
Current or ex‐ smoker	180 (81.4)	134 (80.2)	22 (84.6)	
Unknown	20	—	—	
Diabetes				> 0.9
No	208 (86.3)	156 (86.2)	25 (89.3)	
Yes	7 (2.9)	25 (13.8)	3 (10.7)	
Renal insufficiency				> 0.9
No	234 (97.1)	177 (97.8)	28 (100.0)	
Yes	7 (2.9)	4 (2.2)	0 (0.0)	
Cardiac comorbidity				0.12
No	208 (86.3)	162 (89.5)	22 (78.6)	
Yes	33 (13.7)	19 (10.5)	6 (21.4)	
Respiratory comorbidity				0.14
No	178 (73.9)	134 (74.0)	17 (60.7)	
Yes	63 (26.1)	47 (26.0)	11 (39.3)	
Neoplastic comorbidity				0.7
No	176 (73.0)	136 (75.1)	20 (71.4)	
Yes	65 (27.0)	45 (24.9)	8 (28.6)	
Weight loss				0.6
No	190 (78.8)	144 (79.6)	20 (71.4)	
Yes	51 (21.2)	37 (20.4)	8 (28.6)	
Hospital				0.8
A	32 (13.3)	23 (12.7)	5 (17.9)	
B	86 (35.7)	65 (35.9)	11 (39.3)	
C	29 (12.0)	19 (10.5)	1 (3.6)	
D	58 (24.1)	46 (25.4)	6 (21.4)	
E	36 (14.9)	28 (15.5)	5 (17.9)	
Discussion at a multidisciplinary meeting				> 0.9
No	32 (13.3)	24 (13.3)	3 (10.7)	
Yes	209 (86.7)	157 (86.7)	25 (89.3)	
Supportive care screening tool cited				0.044
No	168 (69.3)	131 (72.4)	15 (53.6)	
Yes	73 (30.7)	50 (27.6)	13 (46.4)	

^a^ NSCLC: Non‐small cell lung cancer.

^b^ SCLC: Small cell lung cancer.

^c^ ECOG: Eastern Co‐operative Oncology Group, Stage unknown patients were excluded for stage‐specific GCT vs non‐GCT, *p*‐value is for assessing association between GCT status and various variables.

For the 209 cases with a documented stage, guideline concordant treatments (GCT) for each stage of disease were received by 181/209 (87%) cases with 28/209 (13%) non‐GCT. Clinical characteristics for these sub‐groups are shown in Table 1. The minority who did not receive GCT were significantly older (*p* = 0.004), with greater ECOG‐PS (*p* < 0.006) and were more likely to have stage III disease (*p* < 0.001). Notably, less than half of the patients diagnosed with stage III disease received GCT, while for the other stages, almost all patients received GCT.

Comparison of VBHC cohort patient characteristics with the general VLCR population recruited from the same health services over the whole 2 annual report years of the study (*n* = 1098) confirmed some significant differences, Table S1. The VBHC cohort contained a higher proportion of people with early‐stage disease (*p* < 0.001) and better ECOG‐PS (*p* = 0.009). The proportion of people with NSCLC (*p* = 0.014), smoking status as a never‐smoker (*p* < 0.001), absence of weight loss (*p* < 0.001), and MDM discussion (*p* < 0.001) was also higher for our pilot VBHC cohort. One hospital, which had a dedicated research fellow at the start of the study, recruited a higher proportion of cases into the cohort (*p* < 0.001).

### 3.3 PROMs/PREMs Response Rates

In total 490 invitations were sent to eligible VLCR participants, and 241 (49%) people responded to the first PROMs/PREMs survey, although not all 241 patients completed all survey items, Figure 1. Overall response rates for subsequent PROMs/PREMs surveys ranged from 42% to 77%, Table 2. A number of challenges were encountered. There was an administrative burden for the registry associated with checking on health status before sending the SMS for subsequent surveys and some timepoints were missed. Some participants missed responding to a survey timepoint but then completed a subsequent survey. Some participants opted to complete the final PREMs but not a third or fourth PROMs linked by the same SMS message. Anecdotally we observed that one hospital with a research fellow approaching patients achieved a higher baseline response rate than the impersonal central approach available to the other pilot hospitals, with no significant difference between all five hospitals' response rates when all sent by central SMS for subsequent surveys (*p* = 0.7).

**TABLE 2:** PROMs and PREMs Survey time‐points and completion rates.

Survey type	Overall	
	Surveys sent (*n*)	Surveys completed (*n*, %)
Baseline PROMs^a^	490	241 (49)
Second PROMs^b^	238	183 (77)
Third PROMs^c^	211	140 (66)
Fourth PROMs^d^	186	78 (42)
Baseline PREMs^a^	490	234 (48)
Final PREMs^d^	233	132 (57)

Abbreviations: PREMS, patient reported experience measures; PROMS, patient reported outcome measures.

^a^ Baseline (around the time of diagnosis).

^b^ 3 months post‐baseline (at the conclusion of first‐line treatment).

^c^ 6 months post‐baseline.

^d^ 9 months post‐baseline.

### 3.4 PROMs

**FIGURE 2:**
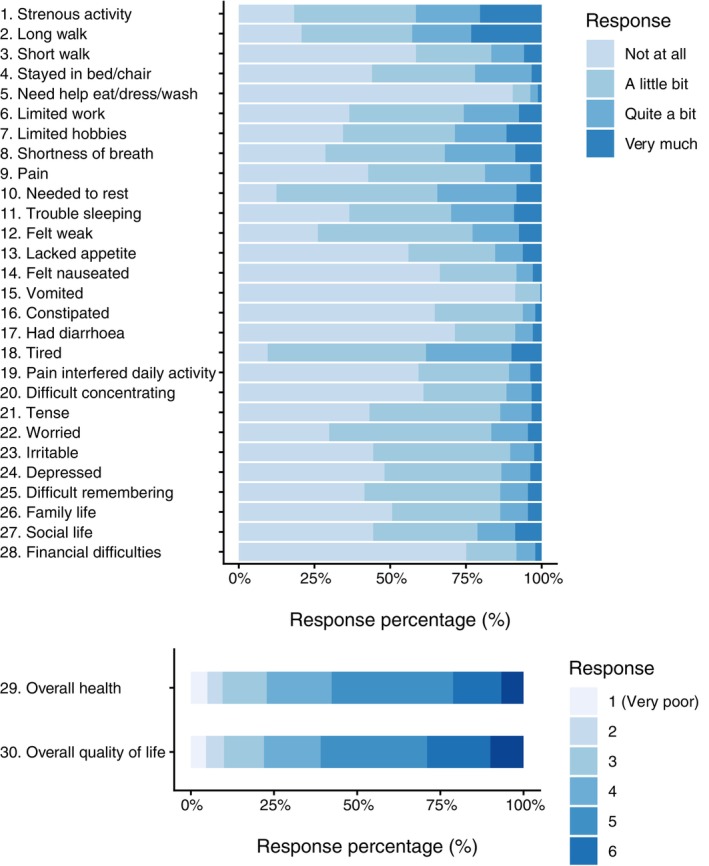
Baseline PROMS: Responses for each of the 30 items in the EORTC QLQ‐C30 Health‐related quality of life questionnaire regarding the impact of health problems in the week prior to questionnaire completion.

Responses to the baseline QLQ‐C30 PROMs are summarized in Figure 2. Subsequent PROMs responses are in Table S3 (QLQ‐C30 items) and Table S6 (QLQ‐LC29 items).

**FIGURE 3:**
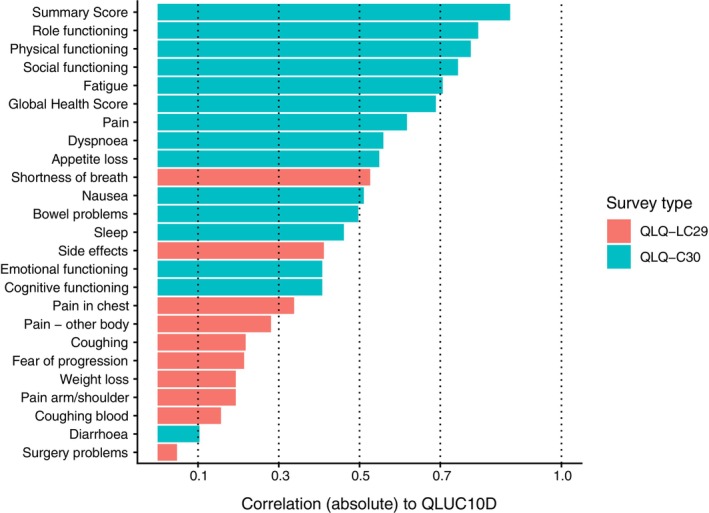
Correlation of EORTC QLU‐C10D preference‐based health utility scores with EORTC QLQ‐C30 and QLQ‐LC29 health‐related quality of life domain scores.

The correlations between the preference‐based HRQL utility scores (i.e., QLU‐C10D scores based on 10 domains of the QLQ‐C30: physical functioning [mobility], role functioning, social functioning, emotional functioning, pain, fatigue, sleep, appetite, nausea, bowel problems [constipation, diarrhea]) and the 15 domains of the QLQ‐C30 and 10 lung cancer‐specific domains of QLQ‐LC29 are shown in Figure 3. The QLU‐C10D scores correlated strongly (> 0.90) with the QLQ‐C30 summary score and several of the QLQ‐C30 functioning scores, which is to be expected given their overlapping content. Correlation with the QLQ‐C30 Global Health/Quality of Life score was 0.70; this is notable, given the two scores have no overlapping content. Correlations with symptoms assessed by the QLQ‐C30 were generally moderate while correlations with EORTC QLQ‐LC29 scores were generally weak, with the exceptions of shortness of breath, side effects and pain in chest, which were moderately correlated with the QLU‐C10D. There were no significant differences in the mean QLU‐C10D scores across the four PROM assessment timepoints (Figure S1).

### 3.5 PREMs

The majority of participants were either very satisfied or satisfied with their overall care at the time of the baseline survey (92%, 216/234), and at the final survey (96%, 127/132). Overall reported satisfaction was so globally high that it did not vary significantly by cancer stage (*p* = 0.7), by receipt of GCT (*p* = 0.2) or by type of health service (*p* = 0.1), and so we did not interrogate this for further associations. Full PREMs responses are shown in Tables S4 and S5.

Regarding the co‐designed PREMs questions that were specifically included due to their importance and value to our consumer groups: 96% participants felt they had access to the best available treatments, 98% had confidence in their treating team, 90% felt they had been treated with respect, 61% felt they had been involved as much as they wanted in decision‐making, and 59% reported that they had been given details for a lung cancer nurse specialist (LCNS). A total of 87% reported that they had come to terms with their lung cancer diagnosis and 91% felt they had adequate support from their friends and family while 61% felt that their family and careers' needs and concerns had been met. A total of 70% reported that they were able to enjoy life and do the things they would usually do for fun.

### 3.6 Survival

**FIGURE 4:**
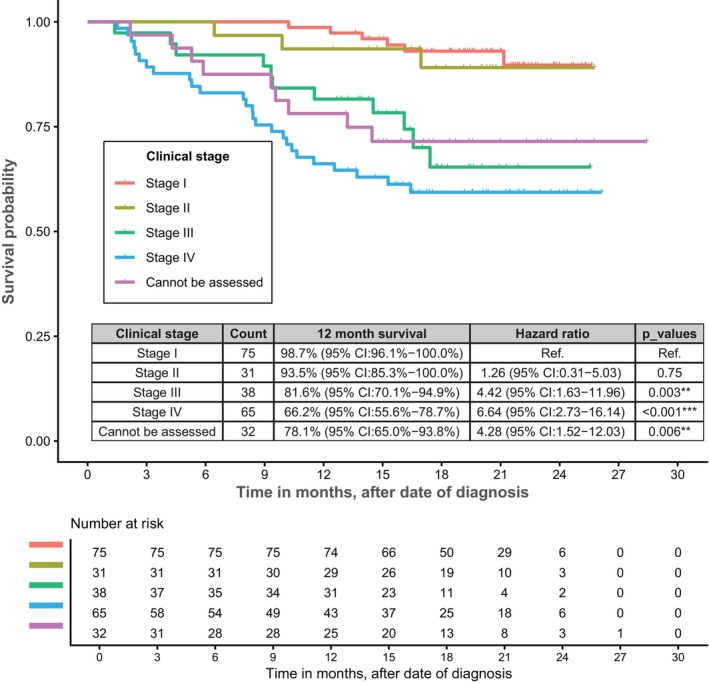
Kaplan‐Meir survival curves by cancer stage at baseline.

One‐year overall survival for the cohort was 83.8% (95% CI, 79.3%–88.6%), Figure S2, and varied significantly by cancer stage: Figure 4. As expected, one year overall survival was highest for early stage disease and lowest for advanced disease: stage I, 98.7% (95% CI, 96.1%–100.0%); stage II, 93.5% (95% CI, 85.3%–100.0%); stage III, 81.6% (95% CI, 70.1%–94.9%); and stage IV, 66.2% (95% CI, 55.6%–78.7%). There was no statistical difference in survival by health service.

### 3.7 Estimated Health System Costs

The estimated cost of Australian optimal lung cancer care by stage during the first year of treatment in AUD (2021) was $29 167, $34 709, $35 757, and $37 913, respectively, for stages I, II, III, and IV, indicating incrementally higher costs in later stages of disease, Table 3. Sensitivity analysis using cost ratios by stage based on more contemporaneous treatments in lung cancer indicated a similar pattern, albeit with higher costs in stages III and IV reflecting the increased costs of disease‐changing immunotherapy and targeted treatments available since 2013.

**TABLE 3:** Estimated health system costs.

Clinical stage at diagnosis	Reviewed studies ($)						Analysis	
	A	B	C	D	E	F	Primary C applying F to estimate costs for Stages I and II (2021 AUD)	Sensitivity C applying F for all stages (2021 AUD)
	Goldsbury 2020^a^	Goldsbury 2020^b^‐1 year in 2013 AUD	Goldsbury 2020^c^‐1 year in 2021 AUD	Buja 2020^d^ EUROS	Buja 2020^e^ AUD	Buja 2020^f^ cost ratio vs. Stage 1		
I	51 531	27 679	31 938	16 291	26 180	1	29 167	29 167
II				19 530	31 385	1.19	34 709	34 709
III	57 905	30 989	35 757	21 938	35 254	1.34	35 757	39 084
IV	54 543	32 857	37 913	22 175	35 635	1.36	37 913	39 667
Unknown	36 462	10 859	12 530	—	—	—	—	—

^a^ Diagnosed between 2006 and 2013 on 45 and up study 1 year before diagnosis and up to 3 years post diagnosis.

^b^ Initial treatment phase (approx. 1 year as per interpretation by Behar Harpaz 2022). Costs were reported in 2013 AUD.

^c^ Inflated to AUD 2021 using Australian Bureau of Statistics. Consumer price index: March Quarter 2024. Canberra, Australia.

^d^ 1‐year costs, Italian health system perspective (in 2019 EUROS). Has applied a more contemporaneous treatment algorithm.

^e^ Converted to AUD (2019 Exchange rates: 1.607).

^f^ In our analyses we assumed the cost ratios by stage remained constant between 2019 and 2021.

### 3.8 Correlation of HRQL With GCT, Survival and Clinical Variables

Given the high degree of correlation among the various PROMs scores and their stability over the study period (reported above), we selected baseline QLU‐C10D utility scores for linear regression analysis to identify clinical and demographic variables that were correlated with HRQL, Table 4. On univariable analysis, HRQL scores were associated with cancer stage (Stage III *p* < 0.001, Stage IV, *p* < 0.002), ECOG performance status ≥ 2 (*p* < 0.001), receipt of GCT (*p* < 0.001), documented respiratory comorbidity at diagnosis (*p* < 0.001), renal insufficiency (*p* = 0.03), cardiac comorbidity (*p* = 0.004), and weight loss (*p* < 0.001). HRQL scores were not associated with age, sex, smoking status, type of lung cancer, discussion at a multi‐disciplinary meeting, or hospital attended.

**TABLE 4:** Factors associated with QLU‐C10D preference‐based health utility scores in linear regression.^a^

Variable	Univariable analysis		Multivariable analysis (*n* = 209)	
	ẞ‐coefficient^b^ (95% CI)	*p*	ẞ‐coefficient^b^ (95% CI)	*p*
Age				
< 60	Reference		Reference	
60–69	−0.03 (−0.12, 0.07)	0.6	0.04 (−0.05, 0.13)	0.3
70–79	0.05 (−0.04, 0.14)	0.3	0.1 (0.02, 0.19)	**0.0018**
80 years and over	0.02 (−0.1, 0.13)	0.8	0.1 (−0.01, 0.21)	0.076
Gender				
Female	Reference			
Male	0 (−0.07, 0.06)	0.9		
Cancer type				
NSCLC	Reference			
SCLC	−0.01 (−0.18, 0.17)	> 0.9		
Stage at diagnosis				
Stage I	Reference		Reference	
Stage II	−0.02 (−0.11, 0.07)	0.7	0.02 (−0.07, 0.1)	0.7
Stage III	−0.16 (−0.24, −0.07)	< 0.001	−0.05 (−0.15, 0.04)	0.3
Stage IV	−0.12 (−0.19, −0.04)	< 0.002	−0.09 (−0.16, −0.02)	**0.017**
Smoking status				
Never smoked	Reference			
Current or ex‐smoker	−0.05 (−0.13, 0.03)	0.2		
Unknown	0.09 (−0.04, 0.22)	0.2		
ECOG status				
0–1	Reference		Reference	
≥ 2	−0.24 (−0.36, −0.12)	< 0.001	−0.13 (−0.25, −0.01)	**0.041**
Unknown	−0.02 (−0.09, 0.06)	0.7	−0.02 (−0.09, 0.05)	0.5
Diabetes				
No	Reference		Reference	
Yes	−0.07 (−0.16, 0.02)	0.13	−0.07 (−0.16, 0.01)	0.088
Renal insufficiency				
No	Reference		Reference	
Yes	−0.34 (−0.56, −0.12)	0.003	−0.21 (−0.43, 0.02)	0.070
Cardiac comorbidity				
No	Reference		Reference	
Yes	−0.14 (−0.23, −0.04)	0.004	−0.12 (−0.21, −0.04)	**0.006**
Respiratory comorbidity				
No	Reference		Reference	
Yes	−0.11 (−0.18, −0.05)	< 0.001	−0.08 (−0.15, −0.02)	**0.014**
Neoplastic comorbidity				
No	Reference			
Yes	0.04 (−0.03, 0.11)	0.3		
Weight loss				
No	Reference		Reference	
Yes	−0.13 (−0.21, −0.06)	< 0.001	−0.07 (−0.14, −0.01)	**0.036**
Hospital				
A	Reference			
B	0.02 (−0.08, 0.12)	0.7		
C	−0.05 (−0.18, 0.08)	0.5		
D	−0.04 (−0.14, 0.07)	0.5		
E	−0.1 (−0.22, 0.01)	0.077		
Discussed at multidisciplinary meeting				
No	Reference		Reference	
Yes	0.04 (−0.05, 0.13)	0.4	0.08 (−0.01, 0.17)	0.069
Supportive care screening completed				
No	Reference			
Yes	−0.06 (−0.12, 0.01)	0.10		
Guideline concordant treatment				
Received GCT	Reference		Reference	
Non GCT received	−0.17 (−0.25, −0.08)	< 0.001	−0.11 (−0.21, 0)	**0.041**

*Note:* Adjusted R‐squared value for multivariable regression = 0.24.

Abbreviations: CI, confidence interval; GCT, guideline concordant treatment.

^a^ Variables that were statistically significant (*p* < 0.05) are bolded.

^b^ ẞ‐coefficient > 0 indicates higher HRQL score (better quality of life) compared to the reference group.

^c^ ẞ‐coefficient < 0 indicates lower HRQL score (lower quality of life) compared to the reference group.

On multivariable analysis, the following subgroups of patients had worse HRQL: those not receiving GCT (*p* = 0.041), Stage IV cancer (*p* = 0.017; reference = Stage 1), ECOG performance status ≥ 2 (*p* = 0.041), and documented respiratory comorbidity at diagnosis (*p* = 0.014), cardiac comorbidity (*p* = 0.006), and weight loss (*p* = 0.036).

Univariable and multivariable regression of QALYs, derived from serial QLU‐C10D and survival data, yielded somewhat similar patterns of associations with clinical variables, Table S2. On multivariable analysis, the following subgroups had lower QALYs: Stage IV cancer (*p* = 0.015), ECOG performance status ≥ 2 (*p* = 0.002), weight loss at presentation (*p* = 0.049), and not receiving GCT (*p* = 0.007).

### 3.9 Registry Dashboard Summarizing Patient‐Centered Value‐Based Lung Cancer Care

**FIGURE 5:**
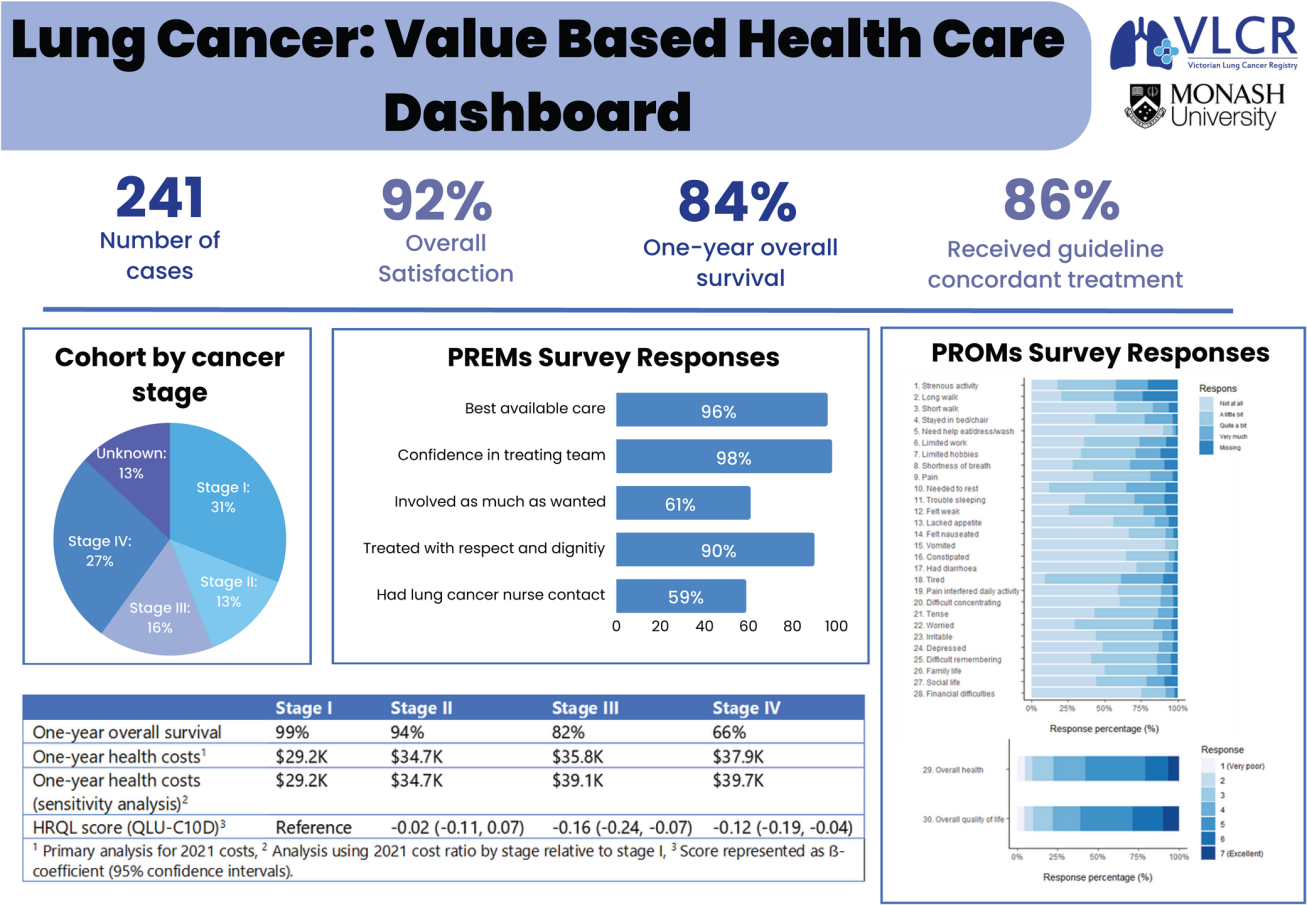
Registry dashboard for health service performance.

We explored combining key metrics that would be relevant for clinicians and informative for health service managers into a dashboard as a proposed template for providing a visual summary of their data, Figure 5. By including cancer stage and survival data with PROMs/PREMs, clinical indicators, and HRQL and health system costs by stage of disease, this dashboard template is intended for future use by the registry to present both population‐level and health‐service specific level real‐time data. This may assist with identifying and developing quality improvement initiatives that add value and drive patient‐centered health outcomes measured for each stage of disease. The dashboard visually highlights how people diagnosed with stage I disease not only have the lowest health system costs; they also have the best overall survival and report significantly better HRQL.

## 4 Discussion

### 4.1 Interpretation of Findings

Our novel aim for this pilot study was to develop a value‐based healthcare report dashboard for our lung cancer registry by combining the introduction of PROMs/PREMs collection with updated health system costs for optimal treatments and our routinely reported clinical outcome measures to identify value‐based, patient‐centered quality improvements at the population level.

#### 4.1.1 Primary Objectives

Our main findings were an overall 49% response rate for baseline PROMs/PREMs surveys sent by the registry, and in the responding cohort, over the timeframe of our study, global health state was good and patient satisfaction was high. Preference‐based HRQL correlated highly with PROMs QLQ30 summary scores and functioning domain scores and did not change over time. We estimated that Australian health system costs for optimal lung cancer treatment increased for stage III and IV disease relative to early stage disease over the last decade.

#### 4.1.2 Secondary Objectives

We found that participants with earlier stages of lung cancer reported better HRQL. Better HRQL was also associated with better ECOG‐PS. Importantly, participants who received GCT also reported better HRQL, even in multivariable analyses that accounted for a wide range of clinical variables including stage of disease.

The overall 49% baseline response rate for PROMs/PREMs was lower than we had estimated and we extended our study timeline to account for this. This response rate is in keeping with PROMs response rates reported by the Australian prostate registry, which ranged between 35% and 76% across eight jurisdictions [20]. Recruitment at baseline was significantly higher for one hospital when there was a dedicated research fellow personally inviting people to participate for several months, compared to hospitals without this facility (77% vs. 40%) with similar subsequent survey response rates at all hospitals. Serial PROMs/PREMs response rates over one year varied in our study from 42% to 77%. A recent review identified 75% PROM response rates in broad registry‐based reports at baseline falling to 50% at 2–5 years [49]. The study however had significant limitations as response rates were not uniformly calculated and reported across all sources. A second review of PROM response rates found 71% at baseline decreasing to 56% at 10 year follow up, with a baseline of 63% for electronic surveys [50]. Completion rates of PROMs in clinical practice in the oncology sector however remain low (< 50%) across multiple settings reflecting a significant difference in disease severity and patient responsiveness [51]. The only other lung cancer registry to have collected PROMs reported a 58% response rate to PROMs collected locally by participating hospitals [22] suggesting our 49% response rate to electronic PROMs for this high burden disease gives us meaningful information on HRQL and could feasibly be extended across our whole registry.

We observed a similar response rate to subsequent surveys, although the total number of responses to serial surveys reduced due to lung cancer progression and because serial PROMs/PREMs requests were only sent to participants who had responded to a prior survey and remained alive on an updated health status check. This might also explain why we found that mean HRQL and overall satisfaction with care did not significantly change over serial surveys, with only those participants whose health remained stable and who continued to engage with the health care system continuing to complete our study surveys. It is well known that people with deteriorating health status and disease progression are more likely to stop replying to surveys, meaning that data that would document their expected deterioration in HRQL is not collected [52].

Among the many PROMs we assessed, we chose to focus on preference‐based HRQL using the EORTC QLU‐C10D (derived from the EORTC QLQ‐C30) for two reasons. First, it covers a broad spectrum of issues, including four key aspects of functioning (mobility plus role, social and emotional functioning) and seven symptoms that cancer patients commonly experience (pain, fatigue, sleep, appetite, nausea, bowel problems (constipation, diarrhea)). Second, its preference‐based scoring incorporates the values of the Australian general population about the relative value of all these issues and survival [46]. Correlation analyses revealed that the QLU‐C10D scores correlated strongly with the QLQ‐C30 global health/quality of life score and summary score, while correlations with symptoms assessed by the QLQ‐C30 were generally moderate, and correlations with EORTC QLQ‐LC29 scores were generally weak. We note that the EORTC QLQ‐LC29 lung‐specific module was designed to capture symptoms of lung cancer and side‐effects of treatment. However, we concluded that, whilst very important, in a registry setting it would be difficult to respond in a timely manner to any issues raised by this module (whether symptoms of disease or treatment), and that these are better placed for use and most importantly rapid response, by treating institutions. So we propose to stop collecting the EORTC QLQ‐LC29 at the registry level moving forward.

We combined serial QLU‐C10D scores and survival data over 1 year to calculate QALYs, which are a key metric for economic analyses and therefore value‐based care. We noted similar patterns in the associations between clinical variables and QLU‐C10D scores versus QALYs, calculated from serial QLU‐C10D scores. This suggests that assessing baseline HRQL (QLU‐C10D) from single timepoint PROMS/PREMs around the time of diagnosis will offer the best value to the registry between information gained, costs of survey coordination and time burden to survey responders. However, further work is needed to confirm this, to include the whole registry.

Our secondary analyses explored how HRQL correlated to routinely collected clinical registry data including receipt of GCT. On multivariable analysis, HRQL was positively associated with earlier stage of lung cancer, better performance status at the time of diagnosis and receipt of GCT for all stages of the disease. Our proposed dashboard visually summarizes these findings. Health system costs for optimal treatments are lower for early stage disease and in our study, HRQL was also positively associated with earlier stage disease, better performance status and receipt of GCT. These findings are not surprising but could be used to guide future value‐based healthcare initiatives for lung cancer, focusing on earlier diagnosis and increased access to GCT to improve health status. With further work, our findings might guide how a value‐based lung cancer care approach could be operationalized, implemented and measured via lung cancer registries.

Overall satisfaction with care received was high at all health services at both diagnosis and after treatment, with 92% and 96% of participants either satisfied or very satisfied with the care they received around diagnosis and treatment respectively, and we did not find any significant associations with our registry clinical data items. We included novel co‐designed unvalidated questions that addressed patient‐centered themes identified as important through a series of consultations with our consumer groups [47]. Shared decision‐making, information, and access to LCNS for support were highly valued in addition to receipt of the best available care (GCT). It was therefore concerning that, despite each health service having a LCNS or research fellow for patient support, only 59% of responders confirmed they had been given this contact information. In this way, our small pilot study identified an aspect of lung cancer care that matters to patients and that needs to be improved. This illustrates how valuable PREMs can be in registries and how they can be used to identify whether and how patient‐centered care can be improved.

### 4.2 Implications for Practice

The main implication for our lung cancer registry based on our pilot feasibility study is that we would gain the highest value from introducing a shorter PROMs/PREMs survey at a single baseline timepoint minimizing patient and administrative burden whilst still obtaining valuable information on HRQL. We have shown that preference‐based HRQL (QLU‐C10D) derived from the EORTC QLQ‐C30 and validated for our Australian population would add valuable information to our reports to health services, with the association of better health state with earlier stage disease at diagnosis, better ECOG PS and receipt of optimal guideline concordant treatment. We plan to validate our baseline custom PREMs survey further. The EORTC QLQ‐LC29 symptom questions are undoubtedly useful when collected at the clinic level for immediate responsive action, but their role at the population level would require clearer definition to justify the additional patient and administrative burden.

For this small pilot study, we used receipt of GCT across the whole cohort as a summarized “distilled” clinical indicator for receipt of optimal treatment, which was defined separately for each stage of lung cancer [32]. People who did not receive GCT were more likely to be in poorer health, older, have stage III NSCLC, have poorer HRQL and shorter survival. Stage III lung cancer is the most heterogeneous of stages, including small primary lung cancers with multiple involved nodes on both sides of the mediastinum to large central primaries with no involved nodes, so it is perhaps not surprising that there is more variation in treatment options, which may need to be individually customized to be optimal. This will only increase with the roll‐out of immunotherapy into the peri‐operative setting as standard of care, joining concurrent chemoradiation with adjuvant immunotherapy for inoperable cases, increasing the range of multi‐modality treatments available for stage III disease. Ideally, with a larger sample size, we might be able to explore stage III outcomes and associations with HRQL and treatment costs in more granular detail.

At the population level, the economic evaluation of lung cancer screening programmes could consider not only the incremental cost‐effectiveness ratio for increasing the proportion of people diagnosed and treated with GCT for early‐stage disease, but also the cost‐utility, that is, taking into account the additional benefit of better HRQL for this early‐stage cohort. This could be prospectively measured by a national lung cancer data platform [53, 54] to show how lung cancer screening impacts both the proportion of people diagnosed with lung cancer at early stage and the HRQL benefits derived by avoiding or delaying the diminished HRQL that comes as the disease progresses.

Irrespective of the stage at the time of lung cancer diagnosis, our findings showed that people who receive GCT report better HRQL. It is also very important for people living with lung cancer to know that they are receiving best available care. In the current rapidly changing field of lung cancer treatment, it is vital that national lung cancer guidelines are regularly updated to ensure that all health services are following current best practice and that detailed granular information on GCT for each stage and subtype of lung cancer, including molecular testing, can be collected by cancer registries.

### 4.3 Strengths and Limitations

This strength of this pilot study and to our knowledge, its unique contribution to the literature, is in setting out a value‐based healthcare framework for quality improvement initiatives by a lung cancer registry. Using PROMs to calculate preference‐based HRQL and exploring how health state is associated with stage, costs and receipt of optimal treatment for lung cancer is entirely novel.

The study does have a number of limitations. Firstly, the electronic survey resulted in selecting people with better ECOG‐PS and earlier stage disease, as highlighted by our post hoc comparison of the study responders to our full registry cohort and we did not have the means to contact people directly regarding their reasons for non‐response. Secondly, this pilot was funded for 5/19 of our health services, all within metropolitan areas. This is a potential source of bias for extrapolating results to the whole registry. It is possible that if PROMs/PREMs collection was introduced across the whole registry, response rates to electronic surveys might be lower for people living in rural and remote locations treated at regional health services.

Due to the relatively small number of participants for each stage and subtype of lung cancer, we used receipt of GCT as a pragmatic and simple global indicator of optimal care by stage to permit correlation with HRQL for the overall cohort. A larger sample size would enable analysis assessing how HRQL relates to optimal treatments by subdivisions of lung cancer stage. Access to more granular data could also assess HRQL stratified by the use of SACT personalized to a panel of molecular tests for individual lung cancers and total dose and fractionation used for delivering radiotherapy. This would be particularly important for stage III lung cancer, where we identified the most variation and where novel peri‐operative multimodality treatments are becoming the new standard of care.

Finally, due to the logistic and ethical difficulties of collecting specific treatment cost data for each individual within our cohort, we used estimated updated Australian health system costs for each stage of lung cancer for our analysis rather than patient‐specific costs. Combining estimated costs with HRQL and clinical outcome data collected by the registry still offers a unique opportunity to identify what might be the most cost‐effective future interventions to improve lung cancer care from the point of view of individuals as well as the health system, for all stages of lung cancer.

## 5 Conclusion

This pilot study demonstrates that incorporating PROMs/PREMs into a clinical quality registry can provide meaningful insight into patient‐centered outcomes in lung cancer. Preference‐based HRQL was associated with cancer stage, performance status, and receipt of guideline concordant treatment, identifying key contributors to value across the care pathway. Integrating HRQL and patient experience with clinical outcomes and estimated stage‐specific costs enabled development of a registry‐level reporting dashboard to inform quality improvement. This framework reinforces the importance of timely diagnosis and delivery of evidence‐based treatment to optimize both survival and quality of life and provides a foundation for broader registry implementation.

## Author Contributions

**Susan V. Harden:** conceptualization, investigation, methodology, supervision, validation, visualization, and writing – original draft. **Madeleine T. King:** conceptualization, data curation, investigation, methodology, validation, writing – review and editing. **Jing Jing Li:** investigation, methodology, validation, writing – original draft, formal analysis, and data curation. **Sanuki Tissera:** data curation, project administration, validation, visualization, writing – review and editing. **Mike Lloyd:** data curation, formal analysis, and methodology. **Lisa Briggs:** investigation, methodology, writing – review and editing. **Tom Wood:** investigation, methodology, writing – review and editing. **Baki Billah:** methodology, validation, formal analysis, and data curation. **Dani Samankula:** data curation and project administration. **Shantelle Smith:** data curation and project administration. **Margaret Brand:** data curation and project administration. **Tali Lang:** investigation, writing – review and editing. **Philip Parente:** investigation, writing – review and editing. **Sarah McGrath:** investigation, writing – review and editing. **David Langton:** investigation, writing – review and editing. **Tegan Dumnall:** investigation, writing – review and editing. **Barton Jennings:** investigation, writing – review and editing. **Sandra Nicholls:** investigation, writing – review and editing. **Rob G. Stirling:** writing – review and editing. **Gary Richardson:** writing – review and editing. **John Zalcberg:** conceptualization, writing – review and editing.

## Conflicts of Interest

John Zalcberg has declared conflicts of interest. The remaining authors declare no conflicts of interest.

## Supporting information


**Data S1:** tca70245‐sup‐0001‐Supinfo.docx.

## Data Availability

The data that support the findings of this study are available on request from the corresponding author. The data are not publicly available due to privacy or ethical restrictions.
