# Supplementary material for: The Association Between Health‐Related Quality of Life Scores and Clinical Outcomes for People Living With Lung Cancer: An Australian Registry Cohort Study Using Patient‐Reported Outcomes to Drive Value‐Based Healthcare
Source: Thorac Cancer. 2026 Feb 23;17(4):e70245. doi: 10.1111/1759-7714.70245 (PMC12962399; doi:10.1111/1759-7714.70245)
Supplement: Supplementary file 1 — Data S1: tca70245‐sup‐0001‐Supinfo.docx. [file TCA-17-e70245-s001.docx]

**SUPPLEMENTARY MATERIAL**

**Supplementary Table 1:** Post-hoc comparison of VBHC cohort (n= 241) and VLCR cohort (n= 1,098).

| **Patient Characteristic** | **VBHC cohort**  n (% of 241) | **VLCR cohort**  n (% of 1098) | **p-value** |
| --- | --- | --- | --- |
| **Age** | | | **0.011** |
| <60 | 40 (16.6) | 133 (12.5) |  |
| 60-69 | 71 (29.5) | 305 (28.7) |  |
| 70-79 | 101 (41.9) | 402 (37.9) |  |
| 80 years and over | 29 (12.0) | 221 (20.8) |  |
| Unknown | 0 | 37 |  |
| **Gender** | | | 0.5 |
| Female | 120 (49.8) | 518 (47.2) |  |
| Male | 121 (50.2) | 580 (52.8) |  |
| **Cancer type** | | | **0.014** |
| NSCLC^1^ | 229 (95.0%) | 988 (90.0) |  |
| SCLC^2^ | 12 (5.0%) | 110 (10.0) |  |
| **Stage at diagnosis** | | | **<0.001** |
| Stage I | 75 (35.9) | 164 (14.9) |  |
| Stage II | 31 (14.8) | 66 (6.0) |  |
| Stage III | 38 (18.2) | 132 (12.0) |  |
| Stage IV | 65 (31.1) | 508 (46.3) |  |
| Unknown | 32 | 228 |  |
| **ECOG^3^ status** | | | **0.009** |
| 0-1 | 160 (88.9) | 653 (80.6) |  |
| ≥2 | 20 (11.1) | 157 (19.4) |  |
| Unknown | 61 | 288 |  |
| **Smoking status** | | | **<0.001** |
| Never smoked | 41 (18.6) | 148 (13.5) |  |
| Current or ex- smoker | 180 (81.4) | 134 (82.7) |  |
| Unknown | 20 | 41 |  |
| **Diabetes** | | | **0.3** |
| No | 208 (86.3) | 915 (83.3) |  |
| Yes | 7 (2.9) | 183 (16.7) |  |
| **Renal insufficiency** | | | 0.8 |
| No | 234 (97.1) | 1,069 (97.4) |  |
| Yes | 7 (2.9) | 29 (2.6) |  |
| **Cardiac comorbidity** | | | 0.7 |
| No | 208 (86.3) | 938 (85.4) |  |
| Yes | 33 (13.7) | 160 (14.6) |  |
| **Respiratory comorbidity** | | | 0.3 |
| No | 178 (73.9) | 775 (70.6) |  |
| Yes | 63 (26.1) | 323 (29.4) |  |
| **Neoplastic comorbidity** | | | 0.2 |
| No | 176 (73.0) | 845 (77.0) |  |
| Yes | 65 (27.0) | 253 (23.0) |  |
| **Weight loss** | | | **<0.001** |
| No | 190 (78.8) | 737 (67.1) |  |
| Yes | 51 (21.2) | 361 (32.9) |  |
| **Hospital** | | | **<0.001** |
| A | 32 (13.3) | 214 (19.5) |  |
| B | 85 (35.3) | 143 (13.0) |  |
| C | 29 (12.0) | 123 (11.2) |  |
| D | 57 (23.7) | 405 (36.9) |  |
| E | 38 (15.8) | 213 (19.4) |  |
| **Discussion at a multidisciplinary meeting** | | | **<0.001** |
| No | 32 (13.3) | 278 (25.3) |  |
| Yes | 209 (86.7) | 820 (74.7) |  |
| **Supportive care screening tool cited** | | | 0.085 |
| No | 168 (69.3) | 698 (63.4%) |  |
| Yes | 73 (30.7) | 400 (36.6%) |  |
| *Variables that were statistically significant (p<0.05) are bolded.* | | | |


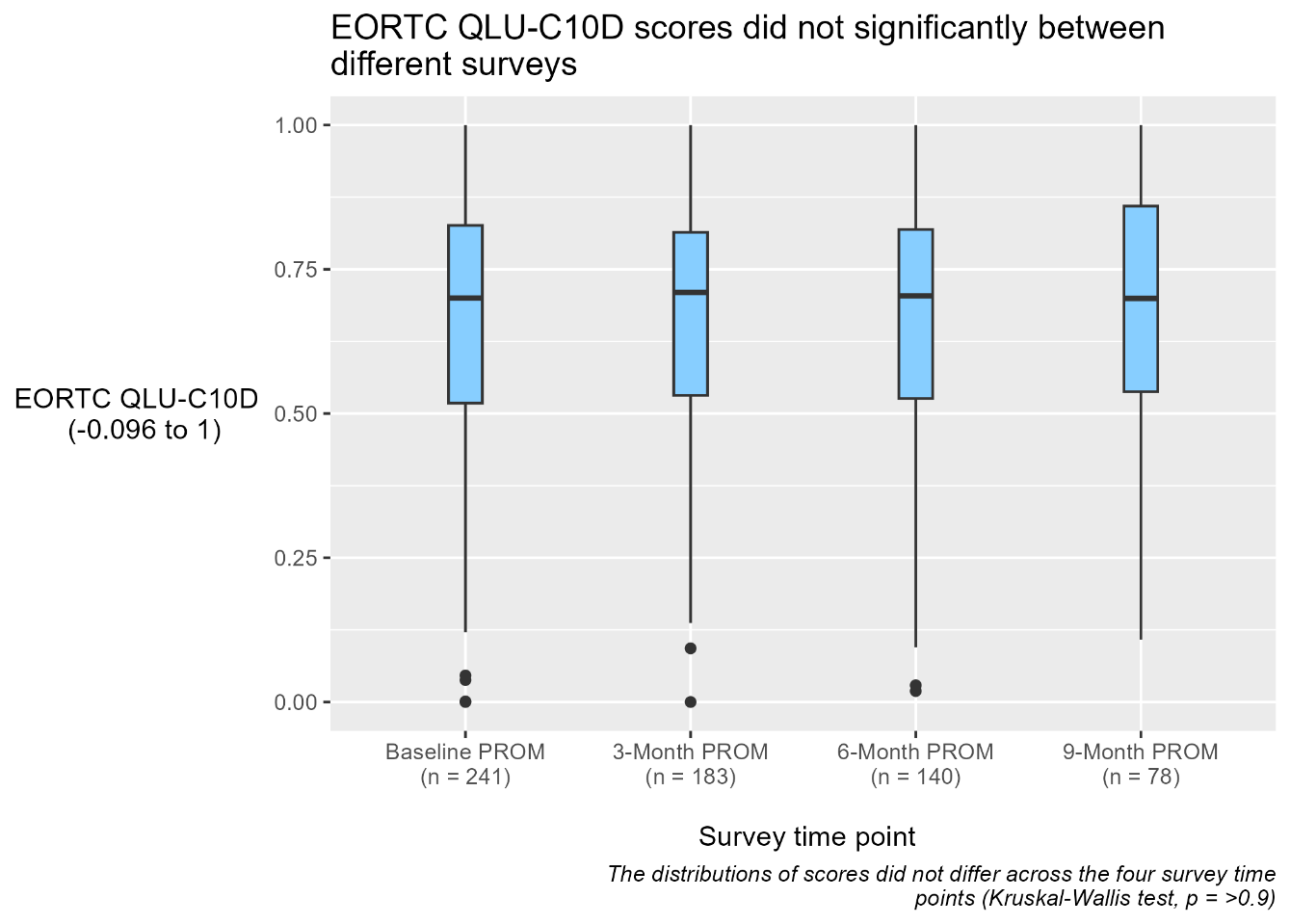
**Supplementary Figure 1:** Box plots of the serial QLU-C10D preference-based health utility scores.

**Supplementary Table 2:** Factors associated with QALYs in linear regression^1^.

| **Variable** | **Univariable analysis** | | **Multivariable analysis (n=209)** | |
| --- | --- | --- | --- | --- |
|  | **ẞ-coefficient^2^ (95% CI)** | **p-value** | **ẞ-coefficient^2^ (95% CI)** | **p-value** |
| **Age** | | | | |
| <60 | Reference | |  |  |
| 60-69 | -0.07 (-0.26, 0.12) | 0.5 |  |  |
| 70-79 | 0 (-0.18, 0.17) | >0.9 |  |  |
| 80 years and over | -0.06 (-0.3, 0.17) | 0.6 |  |  |
| **Gender** | | | | |
| Female | Reference | |  |  |
| Male | -0.04 (-0.16, 0.08) | 0.5 |  |  |
| **Cancer type** | | | | |
| NSCLC | Reference | |  |  |
| SCLC | -0.1 (-0.44, 0.24) | 0.6 |  |  |
| **Stage at diagnosis** | | | | |
| Stage I | Reference | | Reference | |
| Stage II | 0.04 (-0.14, 0.22) | 0.7 | 0.11 (-0.06, 0.29) | 0.2 |
| Stage III | **-0.27 (-0.44, -0.1)** | **0.002** | **-0.07 (-0.27, 0.12)** | 0.5 |
| Stage IV | -0.26 (-0.4, -0.12) | <0.001 | -0.18 (-0.32, -0.03) | **0.015** |
| **Smoking status** | | | | |
| Never smoked | Reference | | Reference | |
| Current or ex-smoker | -0.13 (-0.29, 0.03) | 0.10 | -0.09 (-0.24, 0.06) | 0.2 |
| Unknown | 0.12 (-0.14, 0.38) | 0.4 | 0.09 (-0.16, 0.34) | 0.5 |
| **ECOG status** | | | | |
| 0-1 | Reference | | Reference | |
| ≥2 | -0.47 (-0.71, -0.24) | <0.001 | -0.35 (-0.58, -0.13) | **0.002** |
| Unknown |  |  |  |  |
| **Diabetes** | | | |  |
| No | Reference | | Reference | |
| Yes | -0.14 (-0.32, 0.04) | 0.13 | -0.12 (-0.29, 0.05) | 0.2 |
| **Renal insufficiency** | | | | |
| No | Reference | |  |  |
| Yes | -0.33 (-0.78, 0.11) |  |  |  |
| **Cardiac comorbidity** | | | | |
| No | Reference | |  |  |
| **Yes** | -0.15 (-0.34, 0.04) | 0.12 |  |  |
| **Respiratory comorbidity** | | | | |
| No | Reference | |  |  |
| Yes | -0.13 (-0.26, 0.01) | 0.067 |  |  |
| **Neoplastic comorbidity** | | | | |
| No | Reference | |  |  |
| Yes | 0 (-0.14, 0.14) | >0.9 |  |  |
| **Weight loss** |  |  |  |  |
| No | Reference | | Reference | |
| **Yes** | -0.25 (-0.39, -0.1) | <0.001 | -0.14 (-0.28, 0) | **0.049** |
| **Hospital** |  |  |  |  |
| A | Reference | |  |  |
| B | 0.03 (-0.16, 0.23) | 0.7 |  |  |
| **C** | -0.13 (-0.39, 0.13) | 0.3 |  |  |
| D | -0.01 (-0.21, 0.2) | >0.9 |  |  |
| E | -0.14 (-0.37, 0.09) | 0.2 |  |  |
| **Discussed at multidisciplinary meeting** | | | | |
| No | Reference | |  |  |
| Yes | -0.09 (-0.27, 0.09) | 0.3 |  |  |
| Supportive care screening completed | | | | |
| No | Reference | |  |  |
| Yes | -0.1 (-0.23, 0.03) | 0.113 |  |  |
| **Guideline concordant treatment** | | | | |
| Received GCT | Reference | | Reference | |
| No GCT received | -0.34 (-0.52, -0.17) | <0.001 | -0.27 (-0.47, -0.07) | **0.007** |
| *Adjusted R2 value is 0.19.*  *Abbreviations: Guideline concordant treatment (GCT, confidence interval (CI)*   1. Variables that were statistically significant (p<0.05) are bolded. 2. ẞ-coefficient >0 indicates higher HRQL score (better quality of life) compared to the reference group. 3. ẞ-coefficient <0 indicates lower HRQL score (lower quality of life) compared to the reference group. | | | | |


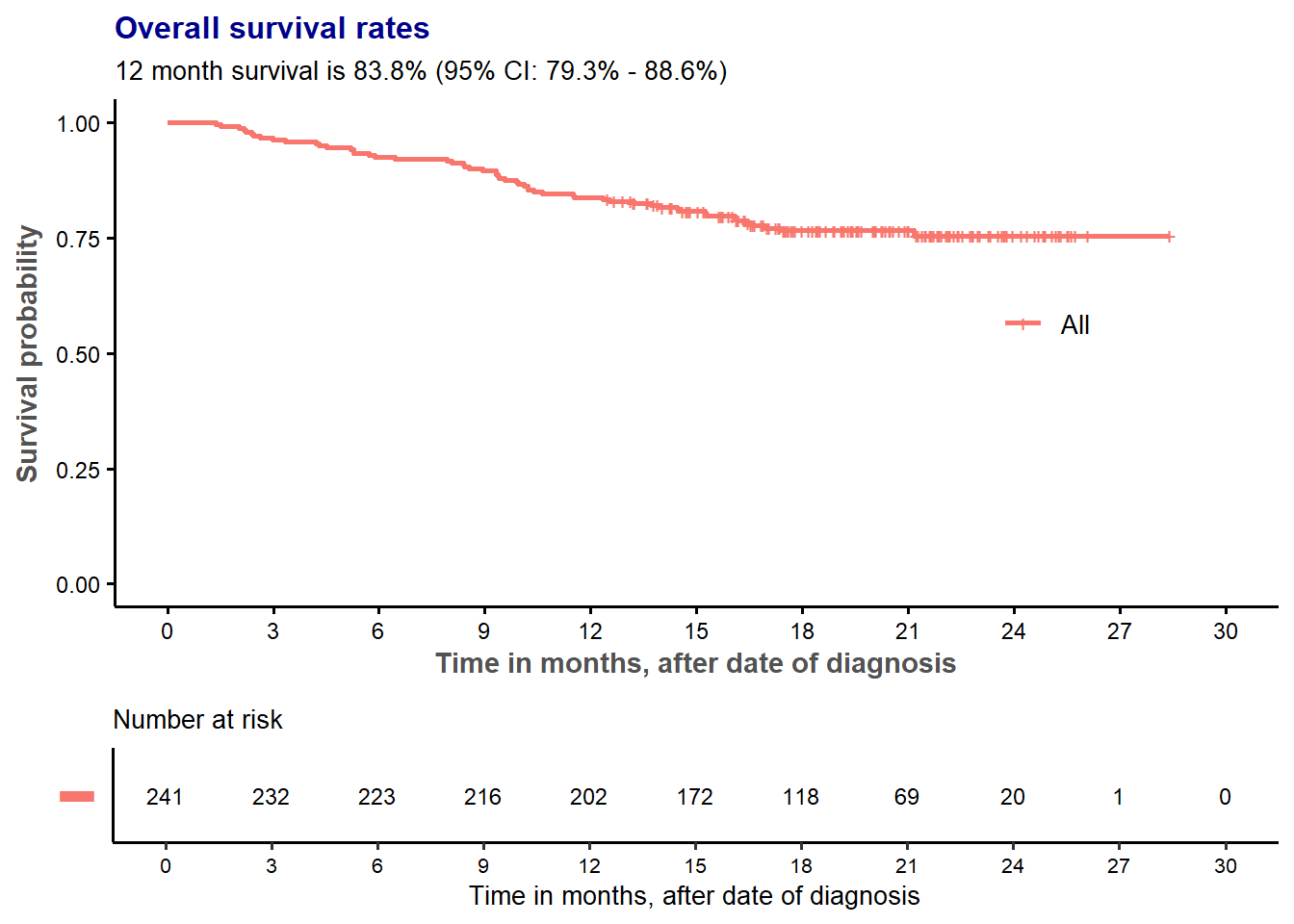
**Supplementary Figure 2:** Kaplan-Meier curve for overall survival.

**Supplementary PROMs/PREMs Appendix**

**PROMs Questionnaire Content and Scoring**

HRQL was assessed with two validated PROMs from the European Organisation for Research and Treatment of Cancer (EORTC) Quality of Life Questionnaire (QLQ) suite: the 30-item core module (QLQ-C30) (1) and the 29-item lung cancer module (QLQ-LC29) (2).

The QLQ-C30 assesses functional health and symptoms commonly experienced by cancer patients regardless of stage and primary cancer site. The QLQ-LC29 contains lung cancer-specific questions, including 24 items relevant to all lung cancer patients and an additional five items specific to surgical patients.

HRQL outcome scores were derived from the QLQ-C30 and QLQ-LC29 data according to EORTC standard scoring procedures (3):

- The QLQ-C30 produced 15 subscales (global health status/quality of life score, five functioning scales (physical, role, social, cognitive and emotional) and eight symptom scores (fatigue, nausea/vomiting, pain, breathlessness, insomnia, appetite loss, constipation, diarrhoea), and financial difficulties(4).
- The QLQ-LC29 produced five multi-item symptom scales (coughing, shortness of breath, side effects, fear of progression, surgery-related symptoms) and five single item scales (coughing blood, pain in the chest, pain in the shoulder, bodily pain, problems with weight loss) (2).
- The QLQ-C30 and QLQ-LC29 scores range from 0 to 100 after linear transformation of the raw scores.
- The direction and interpretation of the scores is as follows:
  - for the global health status/quality of life scale, a high score represents a good quality of life;
  - for the functioning scales, a high score represents a high level of functioning;
  - for the symptom scales, a high score represents a high level of symptoms/problems.

The QLQ-C30 was also summarised as an overall summary score based on 27 of the 30 items (4). The three excluded items were financial concerns, global health status and global quality of life. The summary score ranges from 0 to 100, with a high summary score representing a good quality of life.

Preference-based health utility scores, called EORTC QLU-C10D scores, were also calculated from the 13 of the QLQ-C30 items, covering 10 domains of HRQL data (5): physical functioning (mobility), role functioning, social functioning, emotional functioning, pain, fatigue, sleep, appetite, nausea, bowel problems (constipation, diarrhoea). We used the Australian EORTC QLU-C10D utility algorithm (6), which yielded health utility scores reflecting the preferences of the Australian population. These are called QLU-C10D scores; they have a maximum of 1 (the value of full health); are anchored at zero (dead); health states valued worse than death score less than zero; the value of the worst health state is -0.096. Serial QLU-C10D responses were used to calculate quality adjusted life years (QALYs).

**References**

1. Aaronson NK, Ahmedzai S, Bergman B, Bullinger M, Cull A, Duez NJ, et al. The European Organization for Research and Treatment of Cancer QLQ-C30: a quality-of-life instrument for use in international clinical trials in oncology. J Natl Cancer Inst. 1993;85(5):365-76.

2. Koller M, Shamieh O, Hjermstad MJ, Hornslien K, Young T, Chalk T, et al. Psychometric properties of the updated EORTC module for assessing quality of life in patients with lung cancer (QLQ-LC29): an international, observational field study. Lancet Oncol. 2020;21(5):723-32.

3. Fayers PM, Aaronson NK, Bjordal K, Groenvold M, Curran D, Bottomley A, et al. The EORTC QLQ-C30 Scoring Manual. Brussels: European Organisation for Research and Treatment of Cancer (EORTC); 2001 [cited 15 Feb 2018. Available from: <https://www.eortc.org/app/uploads/sites/2/2018/02/SCmanual.pdf>.

4. Giesinger JM, Kieffer JM, Fayers PM, Groenvold M, Petersen MA, Scott NW, et al. Replication and validation of higher order models demonstrated that a summary score for the EORTC QLQ-C30 is robust. J Clin Epidemiol. 2016;69:79-88.

5. King MT, Costa DS, Aaronson NK, Brazier JE, Cella DF, Fayers PM, et al. QLU-C10D: a health state classification system for a multi-attribute utility measure based on the EORTC QLQ-C30. Qual Life Res. 2016;25(3):625-36.

6. King MT, Viney R, Simon Pickard A, Rowen D, Aaronson NK, Brazier JE, et al. Australian Utility Weights for the EORTC QLU-C10D, a Multi-Attribute Utility Instrument Derived from the Cancer-Specific Quality of Life Questionnaire, EORTC QLQ-C30. Pharmacoeconomics. 2018;36(2):225-38.

**Supplementary Table 3:** Post-baseline PROMs survey responses for each of the 30 items in the EORTC QLQ-C30 health-related quality of life questionnaire

| **Question** | **Question response** | **Second*, n=234 [n (%)]** | **Third*, n=140 [n (%)]** | **Final* n=78 [n (%)]** |
| --- | --- | --- | --- | --- |
| Q1. Do you have any trouble doing strenuous activities, like carrying a heavy shopping bag or a suitcase? | Very much | 27 (14.6) | 22 (15.6) | 13 (16.7) |
|  | Quite a bit | 41 (22.2) | 35 (24.8) | 16 (20.5) |
|  | A little | 81 (43.8) | 60 (42.6) | 33 (42.3) |
|  | Not at all | 36 (19.5) | 24 (17) | 16 (20.5) |
| Q2. Do you have any trouble taking a long walk? | Very much | 31 (16.8) | 22 (15.6) | 13 (16.7) |
|  | Quite a bit | 42 (22.7) | 37 (36.2) | 26 (33.3) |
|  | A little | 73 (39.5) | 58 (41.1) | 23 (29.5) |
|  | Not at all | 39 (21.1) | 24 (17) | 16 (20.5) |
| Q3. Do you have any trouble taking a short walk outside of the house? | Very much | 11 (5.9) | 6 (4.3) | 1 (1.3) |
|  | Quite a bit | 11 (5.9) | 12 (8.5) | 10 (12.8) |
|  | A little | 46 (24.9) | 44 (31.2) | 24 (30.8) |
|  | Not at all | 117 (63.2) | 79 (56) | 43 (55.1) |
| Q4. Do you need to stay in bed or a chair during the day? | Very much | 6 (3.2) | 5 (3.5) | 2 (2.6) |
|  | Quite a bit | 37 (20) | 16 (11.3) | 12 (15.4) |
|  | A little | 50 (27) | 63 (44.7) | 24 (30.8) |
|  | Not at all | 92 (49.7) | 57 (40.4) | 40 (51.3) |
| Q5. Do you need help with eating, dressing, washing yourself or using the toilet? | Very much | 1 (0.5) | 0 (0) | 0 (0) |
|  | Quite a bit | 3 (1.6) | 3 (2.1) | 0 (0) |
|  | A little | 12 (6.5) | 8 (5.7) | 3 (3.8) |
|  | Not at all | 169 (91.4) | 130 (92.2) | 75 (96.2) |
| Q6. During the past week, were you limited in doing either your work or other daily activities? | Very much | 19 (10.3) | 12 (8.5) | 9 (11.5) |
|  | Quite a bit | 28 (15.1) | 33 (23.4) | 15 (19.2) |
|  | A little | 79 (42.7) | 59 (41.8) | 24 (30.8) |
|  | Not at all | 59 (31.9) | 37 (26.2) | 30 (38.5) |
| Q7. During the past week, were you limited in pursuing your hobbies or other leisure time activities? | Very much | 26 (14.1) | 17 (12.1) | 9 (11.5) |
|  | Quite a bit | 21 (11.4) | 27 (19.1) | 15 (19.2) |
|  | A little | 71 (38.4) | 50 (35.5) | 21 (26.9) |
|  | Not at all | 67 (36.2) | 47 (33.3) | 33 (42.3) |
| Q8. During the past week, were you short of breath? | Very much | 14 (7.6) | 11 (7.8) | 5 (6.4) |
|  | Quite a bit | 38 (20.5) | 25 (17.7) | 20 (25.6) |
|  | A little | 90 (48.6) | 71 (50.4) | 33 (42.3) |
|  | Not at all | 43 (23.2) | 34 (24.1) | 20 (25.6) |
| Q9. During the past week, have you had pain? | Very much | 9 (4.9) | 8 (5.7) | 9 (11.5) |
|  | Quite a bit | 24 (13) | 16 (11.3) | 6 (7.7) |
|  | A little | 78 (42.2) | 56 (39.7) | 30 (38.5) |
|  | Not at all | 74 (40) | 61 (43.3) | 33 (42.3) |
| Q10. During the past week, did you need to rest? | Very much | 19 (10.3) | 12 (8.5) | 9 (11.5) |
|  | Quite a bit | 48 (25.9) | 43 (30.5) | 19 (24.4) |
|  | A little | 91 (49.2) | 65 (46.1) | 38 (48.7) |
|  | Not at all | 27 (14.6) | 21 (14.9) | 12 (15.4) |
| Q11. During the past week, have you had trouble sleeping? | Very much | 14 (7.6) | 13 (9.2) | 3 (3.8) |
|  | Quite a bit | 35 (18.9) | 23 (16.3) | 13 (16.7) |
|  | A little | 76 (41.1) | 54 (38.3) | 29 (37.2) |
|  | Not at all | 60 (32.4) | 51 (36.2) | 33 (42.3) |
| Q12. During the past week, have you felt weak? | Very much | 15 (8.1) | 9 (6.4) | 5 (6.4) |
|  | Quite a bit | 27 (14.6) | 27 (19.1) | 14 (17.9) |
|  | A little | 97 (52.4) | 68 (48.2) | 38 (48.7) |
|  | Not at all | 46 (24.9) | 37 (26.2) | 21 (26.9) |
| Q13. During the past week, have you lacked appetite | Very much | 7 (3.8) | 5 (3.5) | 4 (5.1) |
|  | Quite a bit | 24 (13) | 14 (9.9) | 5 (6.4) |
|  | A little | 55 (29.7) | 48 (34) | 19 (24.4) |
|  | Not at all | 99 (53.5) | 74 (52.5) | 50 (64.1) |
| Q14. During the past week, have you felt nauseated? | Very much | 4 (2.2) | 2 (1.4) | 0 (0) |
|  | Quite a bit | 7 (3.8) | 8 (5.7) | 5 (6.4) |
|  | A little | 43 (23.2) | 29 (20.6) | 19 (24.4) |
|  | Not at all | 131 (70.8) | 102 (72.3) | 54 (69.2) |
| Q15. During the past week, have you vomited? | Very much | 0 (0) | 1 (0.7) | 0 (0) |
|  | Quite a bit | 2 (1.1) | 1 (.7) | 2 (2.6) |
|  | A little | 8 (4.3) | 5 (3.5) | 4 (5.1) |
|  | Not at all | 175 (94.6) | 134 (95) | 72 (92.3) |
| Q16. During the past week, have you been constipated? | Very much | 50 (27) | 7 (5) | 3 (3.8) |
|  | Quite a bit | 15 (8.1) | 7 (5) | 5 (6.4) |
|  | A little | 50 (27) | 35 (24.8) | 23 (29.5) |
|  | Not at all | 112 (60.5) | 92 (65.2) | 47 (60.3) |
| Q17. During the past week, have you had diarrhoea? | Very much | 0 (0) | 0 (0) | 0 (0) |
|  | Quite a bit | 4 (2.2) | 4 (2.8) | 4 (5.1) |
|  | A little | 42 (22.7) | 18 (12.8) | 13 (16.7) |
|  | Not at all | 139 (75.1) | 119 (84.4) | 61 (78.2) |
| Q18. During the past week, were you tired? | Very much | 20 (10.8) | 12 (8.5) | 8 (10.3) |
|  | Quite a bit | 53 (28.6) | 47 (33.3) | 21 (26.9) |
|  | A little | 95 (51.4) | 75 (53.2) | 40 (51.3) |
|  | Not at all | 17 (9.2) | 7 (5) | 9 (11.5) |
| Q19. During the past week, did pain interfere with your daily activities? | Very much | 9 (4.9) | 8 (5.7) | 3 (3.8) |
|  | Quite a bit | 17 (9.2) | 9 (6.4) | 8 (10.3) |
|  | A little | 56 (30.3) | 48 (34) | 20 (25.6) |
|  | Not at all | 103 (55.7) | 76 (53.9) | 47 (60.3) |
| Q20. During the past week, have you had difficulty in concentrating on things, like reading a newspaper or watching television? | Very much | 7 (3.8) | 5 (3.5) | 2 (2.6) |
|  | Quite a bit | 13 (7) | 9 (6.4) | 5 (6.4) |
|  | A little | 55 (29.7) | 44 (31.2) | 24 (30.8) |
|  | Not at all | 110 (59.5) | 83 (58.9) | 47 (60.3) |
| Q21. During the past week, did you feel tense? | Very much | 9 (4.9) | 4 (2.8) | 2 (2.6) |
|  | Quite a bit | 16 (8.6) | 16 (11.3) | 7 (9) |
|  | A little | 90 (48.6) | 66 (46.8) | 40 (51.3) |
|  | Not at all | 70 (37.8) | 55 (39) | 29 (37.2) |
| Q22. During the past week, did you worry? | Very much | 11 (5.9) | 7 (5) | 3 (3.8) |
|  | Quite a bit | 21 (11.4) | 25 (17.7) | 14 (17.9) |
|  | A little | 103 (55.7) | 75 (53.2) | 38 (48.7) |
|  | Not at all | 50 (27) | 34 (24.1) | 23 (29.5) |
| Q23. During the past week, did you feel irritable? | Very much | 3 (1.6) | 6 (4.3) | 3 (3.8) |
|  | Quite a bit | 10 (5.4) | 11 (7.8) | 5 (6.4) |
|  | A little | 95 (51.4) | 63 (44.7) | 37 (47.4) |
|  | Not at all | 77 (41.6) | 61 (43.3) | 33 (42.3) |
| Q24. During the past week, did you feel depressed? | Very much | 7 (3.8) | 5 (3.5) | 3 (3.8) |
|  | Quite a bit | 13 (7) | 12 (8.5) | 6 (7.7) |
|  | A little | 80 (43.2) | 62 (44) | 28 (35.9) |
|  | Not at all | 85 (45.9) | 62 (44) | 41 (52.6) |
| Q25. During the past week, have you had difficulty remembering things? | Very much | 4 (2.2) | 3 (2.1) | 1 (1.3) |
|  | Quite a bit | 13 (7) | 14 (9.9) | 9 (11.5) |
|  | A little | 77 (41.6) | 58 (41.1) | 32 (41) |
|  | Not at all | 91 (49.2) | 66 (46.8) | 36 (46.2) |
| Q26. During the past week, has your physical condition or medical treatment interfered with your family life? | Very much | 6 (3.2) | 7 (5) | 2 (2.6) |
|  | Quite a bit | 22 (11.9) | 13 (9.2) | 10 (12.8) |
|  | A little | 64 (34.6) | 41 (29.1) | 22 (28.2) |
|  | Not at all | 93 (50.3) | 80 (56.7) | 44 (56.4) |
| Q27. During the past week, has your physical condition or medical treatment interfered with your social activities? | Very much | 16 (8.6) | 9 (6.4) | 7 (9) |
|  | Quite a bit | 30 (16.2) | 15 (10.6) | 8 (10.3) |
|  | A little | 62 (33.5) | 54 (38.3) | 25 (32.1) |
|  | Not at all | 77 (41.6) | 63 (44.7) | 38 (48.7) |
| Q28. During the past week, has your physical condition or medical treatment caused you financial difficulties? | Very much | 5 (2.7) | 3 (2.1) | 2 (2.6) |
|  | Quite a bit | 6 (3.2) | 6 (4.3) | 4 (5.1) |
|  | A little | 33 (17.8) | 27 (19.1) | 11 (14.1) |
|  | Not at all | 141 (76.2) | 105 (74.5) | 61 (78.2) |
| Q29. How would you rate your health during the past week? | 1 (very poor) | 6 (3.2) | 2 (1.4) | 3 (3.8) |
|  | 2 | 12 (6.5) | 3 (2.1) | 1 (1.3) |
|  | 3 | 17 (9.2) | 18 (12.8) | 15 (19.2) |
|  | 4 | 54 (29.2) | 41 (29.1) | 15 (19.2) |
|  | 5 | 54 (29.2) | 49 (34.8) | 24 (30.8) |
|  | 6 | 33 (17.8) | 22 (15.6) | 13 (16.7) |
|  | 7 (excellent) | 9 (4.9) | 6 (4.3) | 7 (9) |
| Q30. How would you rate your overall quality of life during the past week? | 1 (very poor) | 5 (2.7) | 2 (1.4) | 2 (2.6) |
|  | 2 | 8 (4.3) | 3 (2.1) | 1 (1.3) |
|  | 3 | 22 (11.9) | 17 (12.1) | 14 (17.9) |
|  | 4 | 33 (17.8) | 32 (22.7) | 12 (15.4) |
|  | 5 | 61 (33) | 53 (37.6) | 28 (35.9) |
|  | 6 | 35 (18.9) | 22 (15.6) | 13 (16.7) |
|  | 7 (excellent) | 21 (11.4) | 12 (8.5) | 8 (10.3) |
| **Second refers to 3-month survey, Third refers to 6-month survey, Final refers to 9-month survey* | | | | |

**Supplementary Table 4:** Baseline PREMS survey responses.

| **Question** | **Question response** | **Answer, n=234 [n (%)]** |
| --- | --- | --- |
| Q1. How many times did you see your GP (family doctor) about the symptoms caused by your cancer before you went to hospital? | None – I did not see my GP before going to hospital | 74 (31.6) |
|  | I saw my GP once | 56 (23.9) |
|  | I saw my GP twice | 48 (20.5) |
|  | I saw my GP 3 or 4 times | 35 (15) |
|  | I saw my GP 5 or more times | 21 (9) |
| Q2. How long was it between when you were referred to a specialist hospital clinic and your first appointment? | Less than 2 weeks | 132 (56.4) |
|  | More than 2 weeks but within 4 weeks | 54 (23.1) |
|  | More than a month | 18 (7.7) |
|  | Other | 19 (8.1) |
|  | Don’t know/can’t remember | 11 (4.7) |
| Q3. Did all the health professionals involved in your diagnosis communicate to you with respect and understanding? | Yes always | 178 (76.1) |
|  | Yes most of the time | 33 (14.1) |
|  | Some but not others | 19 (8.1) |
|  | No | 3 (1.3) |
|  | Not sure/cannot remember | 1 (0.4) |
| Q4. When you were told you had lung cancer, did you understand the doctor’s explanation of what was wrong with you? | I understood it completely | 146 (62.4) |
|  | I understood most of it | 58 (24.8) |
|  | I understood some of it | 23 (9.8) |
|  | No, I did not understand it | 5 (2.1) |
|  | Not sure / cannot remember | 2 (0.9) |
| Q5. When you were told about your lung cancer and the treatment plan, were you given information you could understand about the treatment options (e.g. written information or being spoken with)? | Yes, definitely | 157 (67.1) |
|  | Yes, I think so | 57 (24.4) |
|  | No, definitely not | 7 (3) |
|  | No, I do not think so | 11 (4.7) |
|  | Not sure / cannot remember | 2 (0.9) |
| Q6. When you were told you had lung cancer, were you asked if you had any practical, family, emotional, spiritual or physical support needs? | Yes, definitely | 95 (40.6) |
|  | Yes, I think so | 66 (28.2) |
|  | No, definitely not | 21 (9) |
|  | No, I do not think so | 48 (20.5) |
|  | Not sure / cannot remember | 4 (1.7) |
| Q7. When you were told you had lung cancer, were you given information about who you could contact to help support your wellbeing (e.g. referral to an allied health professional, support group, cancer helpline)? | Yes, definitely | 77 (32.9) |
|  | Yes, I think so | 49 (20.9) |
|  | No, definitely not | 35 (15) |
|  | No, I do not think so | 64 (27.4) |
|  | Not sure / cannot remember | 9 (3.8) |
| Q8. Were you given the name and contact details of a Lung Cancer Nurse/Co-ordinator who would be in charge of navigating your care? | Yes, definitely | 101 (43.2) |
|  | Yes, I think so | 36 (15.4) |
|  | No, definitely not | 42 (17.9) |
|  | No, I do not think so | 50 (21.4) |
|  | Not sure / cannot remember | 5 (2.1) |
| Q9. When you were told you had lung cancer, were the needs or concerns of your family and/or carers considered and accommodated/provided for? | Yes, definitely | 75 (32.1) |
|  | Yes, I think so | 67 (28.6) |
|  | No, definitely not | 27 (11.5) |
|  | No, I do not think | 58 (24.8) |
|  | Not sure / cannot remember | 7 (3) |
| Q10. When you were told you had cancer, if you were a current smoker or recent quitter, were you offered help to stop smoking? | Yes, and I did stop smoking | 35 (15) |
|  | Yes, but I didn’t choose to take this | 6 (2.6) |
|  | I wasn't a current smoker or recent quitter | 32 (13.7) |
|  | No and I didn’t want any | 11 (4.7) |
|  | No, I wasn’t a current smoker or recent quitter | 126 (53.8) |
|  | No but I would have liked to be offered help | 17 (7.3) |
|  | Not sure / cannot remember | 7 (3) |
| Q11. Lung cancer can affect anybody. Did you feel that you were asked too often about smoking and/or made to feel guilty about it? | Yes, often | 17 (7.3) |
|  | Yes, occasionally | 38 (16.2) |
|  | No, definitely not | 100 (42.7) |
|  | No, I do not think so | 77 (32.9) |
|  | Not sure/cannot remember: | 2 (0.9) |
| Q12. Were you involved as much as you wanted to be in decisions about your care and treatment? | Yes, definitely | 143 (61.1) |
|  | Yes, to some extent | 54 (23.1) |
|  | Only one type of suitable treatment | 14 (6) |
|  | No | 22 (9.4) |
|  | Not sure / cannot remember | 1 (0.4) |
| Q13. Have you been able to accept your lung cancer diagnosis? | Yes, definitely | 153 (65.4) |
|  | Yes, to some extent | 51 (21.8) |
|  | Somewhat | 20 (8.5) |
|  | A little bit | 8 (3.4) |
|  | Not at all | 2 (0.9) |
| Q14. Are you able to get sufficient emotional and psychological support from your friends and family? | Yes, definitely | 174 (74.4) |
|  | Yes, to some extent | 38 (16.2) |
|  | Somewhat | 11 (4.7) |
|  | A little bit | 4 (1.7) |
|  | Not at all | 7 (3) |
| Q15. Are you content with your Quality of Life right now? | Yes, definitely | 82 (35) |
|  | Yes, to some extent | 76 (32.5) |
|  | Somewhat | 36 (15.4) |
|  | A little bit | 16 (6.8) |
|  | Not at all | 24 (10.3) |
| Q16. Overall, how satisfied were you with the care you received from the health professionals involved in your lung cancer diagnosis? | Very satisfied | 150 (64) |
|  | Satisfied | 66 (28) |
|  | Neither | 12 (5) |
|  | Dissatisfied | 4 (1.5) |
|  | Very dissatisfied | 4 (1.5) |
| Q17. Please tell us if you are... (tick all boxes that apply) | Completing the survey as a patient | 114 (48.7) |
|  | Willing to be contacted to complete a similar survey in 3 months | 106 (45.3) |
|  | Completing the survey as a carer, on behalf of a patient | 14 (6) |

**Supplementary Table 5:** Final PREMS survey responses (9 months after baseline).

| **Question** | **Question response** | **Answer, n=132 [n (%)]** |
| --- | --- | --- |
| Q1. Did the health professionals involved in your lung cancer treatment and care treat you with respect and dignity? | Yes, always | 121 (91.7) |
|  | Yes, sometimes | 11 (8.3) |
| Q2. Were you involved as much as you wanted to be in decisions about your lung cancer care and treatment? | Yes, definitely | 94 (71.2) |
|  | Yes, to some extent | 30 (22.7) |
|  | Only one type of suitable treatment | 4 (3) |
|  | No | 4 (3) |
| Q3. Did you have confidence and trust in the health professionals treating you? | Yes, always | 111 (84.1) |
|  | Yes, to some extent | 19 (14.4) |
|  | No | 2 (1.5) |
| Q4. Did you feel that you had access to the best available treatments, tests and investigations at the time? | Yes, definitely | 107 (81.1) |
|  | Yes, to some extent | 20 (15.2) |
|  | No | 3 (2.3) |
|  | Not sure/cannot remember | 2 (1.5) |
| Q5. Did any member of your health care team discuss with you the possibility of you taking part in cancer research or clinical trials? | Yes, and I was happy for them to do this | 49 (37.1) |
|  | Yes, but I was not happy for them to do this | 2 (1.5) |
|  | No, but I would have liked them to have done this | 41 (31.1) |
|  | No, and I am glad they did not do this | 14 (10.6) |
|  | Not sure/cannot remember | 26 (19.7) |
| Q6. Were you given the name of a Clinical Nurse Specialist or coordinator to help you navigate your care pathway? | Yes, definitely | 46 (34.8) |
|  | Yes, I think so | 23 (17.4) |
|  | No, definitely not | 25 (18.9) |
|  | No, I do not think so | 29 (22) |
|  | Not sure/cannot remember | 9 (6.8) |
| Q7. Throughout your cancer care and treatment, has there been a health professional or a team of health professionals you could contact if you had any questions about your care? | Yes, at least one | 90 (68.2) |
|  | Yes, but not all the time | 19 (14.4) |
|  | No | 15 (11.4) |
|  | Not sure/cannot remember | 8 (6.1) |
| Q8. Were you satisfied with how your care team engaged with your family/carers/support people? | Yes, definitely | 66 (50) |
|  | Yes, I think so | 37 (28) |
|  | No, definitely not | 7 (5.3) |
|  | No, I do not think so | 15 (11.4) |
|  | Not sure/cannot remember | 7 (5.3) |
| Q9. Throughout your cancer care, has there been a time when you thought tests or radiology (imaging) examinations were being repeated unnecessarily? | 4 or more times | 1 (0.8) |
|  | 2-3 times | 4 (3) |
|  | Once | 2 (1.5) |
|  | No, never | 124 (93.9) |
|  | Not sure/cannot remember | 1 (0.8) |
| Q10. Were you given: Information about things you could do to keep well and stay healthy (e.g. exercise, diet, mindfulness, stopping smoking)? | I was given this information | 81 (61.4) |
|  | I was not given this information | 18 (13.6) |
|  | I would have liked more | 21 (15.9) |
|  | I am still receiving treatment | 5 (3.8) |
|  | Not sure/cannot remember | 7 (5.3) |
| Q11. Were you given: Information about how to get extra support if you or your family wanted it? | I was given this information | 64 (48.5) |
|  | I was not given this information | 39 (29.5) |
|  | I would have liked more | 15 (11.4) |
|  | Not sure/cannot remember | 14 (10.6) |
| Q12. As far as you know, was your GP kept informed about your condition and your treatment? | Yes | 113 (85.6) |
|  | I do not have a regular GP | 1 (0.8) |
|  | No | 10 (7.6) |
|  | Not sure/cannot remember | 8 (6.1) |
| Q13. Are you still receiving treatment for your lung cancer? | Yes | 68 (51.5) |
|  | No, I did not receive treatment | 13 (9.8) |
|  | No, my treatment is finished | 51 (38.6) |
| Q14. If receiving treatment, how long is your journey from home to the treating hospital? | Under 1 hour | 59 (44.7) |
|  | 1-2 hours | 15 (11.4) |
|  | 2-4 hours | 2 (1.5) |
|  | Over 6 hours | 1 (0.8) |
|  | N/A - not currently receiving treatment | 55 (41.7) |
| Q15. Did the health professionals involved in your care check that you understood about possible side-effects from your current treatment? | Yes, definitely | 51 (38.6) |
|  | Yes, to some extent | 14 (10.6) |
|  | N/A – not currently receiving treatment | 57 (43.2) |
|  | No | 8 (6.1) |
|  | Not sure/cannot remember | 2 (1.5) |
| Q16. If you have finished your cancer treatment were you given a written plan that included information about your follow-up care over the next 12 months | I was given this information | 36 (27.3) |
|  | I was not given this information | 21 (15.9) |
|  | I would have liked more | 8 (6.1) |
|  | N/A – still receiving treatment | 57 (43.2) |
|  | Not sure/cannot remember | 10 (7.6) |
| Q17. How satisfied were you with the overall care you received from all health professionals involved in your treatment? | Very satisfied | 91 (68.9) |
|  | Satisfied | 36 (27.3) |
|  | Neither | 4 (3) |
|  | Dissatisfied | 1 (0.8) |
| Q18. Are you able to enjoy life and the things you usually do for fun? | Yes, definitely | 35 (26.5) |
|  | Yes, to some extent | 57 (43.2) |
|  | Somewhat | 19 (14.4) |
|  | A little bit | 14 (10.6) |
|  | Not at all | 7 (5.3) |
| Q19. Are you content with how you are able to function and manage day to day life (your quality of life) right now? | Yes, definitely | 45 (34.1) |
|  | Yes, to some extent | 50 (37.9) |
|  | Somewhat | 23 (17.4) |
|  | A little bit | 8 (6.1) |
|  | Not at all | 6 (4.5) |
| Q20. Please tell us if you are: | Completing the survey as a patient | 129 (97.7) |
|  | Completing the survey as a carer, on behalf of a patient | 3 (2.3) |

**Supplementary Table 6:** Lung cancer specific PROMS survey: responses for each of the 29 items in the EORTC QLQ-LC29 lung cancer module, which was assessed twice (3 and 6 months after baseline)

| **Question - During the past week:** | **Question response** | **3-month, n=190 [n (%)]** | **6-month, n=130 [n (%)]** |
| --- | --- | --- | --- |
| Q1. Have you coughed? | Not at all | 47 (24.7) | 38 (29.2) |
|  | A little | 91 (47.9) | 64 (49.2) |
|  | Quite a bit | 41 (21.6) | 22 (16.9) |
|  | Very much | 11 (5.8) | 6 (4.6) |
| Q2. Have you coughed up blood? | Not at all | 181 (95.3%) | 124 (95.4%) |
|  | A little | 7 (3.7%) | 6 (4.6%) |
|  | Quite a bit | 2 (1.1%) | 0 (0) |
|  | Very much | 0 (0) | 0 (0) |
| Q3. Have you been short of breath when resting? | Not at all | 112 (58.9) | 83 (63.8) |
|  | A little | 62 (32.6) | 42 (32.3) |
|  | Quite a bit | 14 (7.4) | 3 (2.3) |
|  | Very much | 2 (1.1) | 2 (1.5) |
| Q4. Have you been short of breath when you walked? | Not at all | 38 (20.0) | 25 (19.2) |
|  | A little | 88 (46.3) | 72 (55.4) |
|  | Quite a bit | 47 (24.7) | 22 (16.9) |
|  | Very much | 17 (8.9) | 11 (8.5) |
| Q5. Have you been short of breath when you climbed stairs? | Not at all | 32 (16.8) | 30 (23.1) |
|  | A little | 82 (43.2) | 52 (40.0) |
|  | Quite a bit | 44 (23.2) | 31 (23.8) |
|  | Very much | 32 (16.8) | 17 (13.1) |
| Q6. Have you had a sore mouth or tongue? | Not at all | 150 (78.9) | 100 (76.9) |
|  | A little | 27 (14.2) | 24 (18.5) |
|  | Quite a bit | 8 (4.2) | 5 (3.8) |
|  | Very much | 5 (2.6) | 1 (0.8) |
| Q7. Have you had problems swallowing? | Not at all | 155 (81.6) | 115 (88.5) |
|  | A little | 31 (16.3) | 11 (8.5) |
|  | Quite a bit | 3 (1.6) | 2 (1.5) |
|  | Very much | 1 (0.5) | 2 (1.5) |
| Q8. Have you had tingling hands or feet? | Not at all | 120 (63.2) | 90 (69.2) |
|  | A little | 53 (27.9) | 26 (20.0) |
|  | Quite a bit | 10 (5.3) | 8 (6.2) |
|  | Very much | 7 (3.7) | 6 (4.6) |
| Q9. Have you had hair loss? | Not at all | 141 (74.2) | 86 (66.2) |
|  | A little | 32 (16.8) | 26 (20.0) |
|  | Quite a bit | 10 (5.3) | 9 (6.9) |
|  | Very much | 7 (3.7) | 9 (6.9) |
| Q10. Have you had pain in your chest? | Not at all | 98 (51.6) | 76 (58.5) |
|  | A little | 75 (39.5) | 41 (31.5) |
|  | Quite a bit | 15 (7.9) | 10 (7.7) |
|  | Very much | 2 (1.1) | 3 (2.3) |
| Q11. Have you had pain in your arm or shoulder? | Not at all | 127 (66.8) | 88 (67.7) |
|  | A little | 48 (25.3) | 29 (22.3) |
|  | Quite a bit | 2 (6.3) | 9 (6.9) |
|  | Very much | 3 (1.6) | 4 (3.1) |
| Q12. Have you had pain in other parts of your body? | Not at all | 98 (51.6) | 76 (58.5) |
|  | A little | 75 (39.5) | 41 (31.5) |
|  | Quite a bit | 15 (7.9) | 10 (7.7) |
|  | Very much | 2 (1.1) | 3 (2.3) |
| Q13. Have you had allergic reactions? | Not at all | 165 (86.8) | 115 (88.5) |
|  | A little | 13 (6.8) | 12 (9.2) |
|  | Quite a bit | 8 (4.2) | 2 (1.5) |
|  | Very much | 4 (2.1) | 1 (0.8) |
| Q14. Have you had burning or sore eyes? | Not at all | 139 (73.2) | 95 (73.1) |
|  | A little | 38 (20.0) | 28 (21.5) |
|  | Quite a bit | 10 (5.3) | 7 (5.4) |
|  | Very much | 3 (1.6) | 0 (0) |
| Q15. Have you been dizzy? | Not at all | 117 (61.6) | 76 (58.5) |
|  | A little | 62 (32.6) | 46 (35.4) |
|  | Quite a bit | 9 (4.7) | 8 (6.2) |
|  | Very much | 2 (1.1) | 0 (0) |
| Q16. Have you had splitting fingernails or toenails? | Not at all | 144 (75.8) | 96 (73.8) |
|  | A little | 31 (16.3) | 29 (22.3) |
|  | Quite a bit | 12 (6.3) | 3 (2.3) |
|  | Very much | 3 (1.6) | 2 (1.5) |
| Q17. Have you had skin problems (e.g. itchy, dry)? | Not at all | 81 (42.6) | 53 (40.8) |
|  | A little | 75 (39.5) | 55 (42.3) |
|  | Quite a bit | 22 (11.6) | 15 (11.5) |
|  | Very much | 12 (6.3) | 7 (5.4) |
| Q18. Have you had problems speaking? | Not at all | 163 (85.8) | 114 (87.7) |
|  | A little | 20 (10.5) | 14 (10.8) |
|  | Quite a bit | 3 (1.6) | 1 (0.8) |
|  | Very much | 4 (2.1) | 1 (0.8) |
| Q19. Have you been afraid of tumor progression? | Not at all | 75 (39.5) | 50 (38.5) |
|  | A little | 78 (41.1) | 54 (41.5) |
|  | Quite a bit | 22 (11.6) | 16 (12.3) |
|  | Very much | 15 (7.9) | 10 (7.7) |
| Q20. Have you had thin or lifeless hair as a result of your disease or treatment? | Not at all | 128 (67.4) | 86 (66.2) |
|  | A little | 43 (22.6) | 26 (20.0) |
|  | Quite a bit | 16 (8.4) | 10 (7.7) |
|  | Very much | 3 (1.6) | 8 (6.2) |
| Q21. Have you worried about your health in the future? | Not at all | 32 (16.8) | 20 (15.4) |
|  | A little | 96 (50.5) | 70 (53.8) |
|  | Quite a bit | 35 (18.4) | 22 (16.9) |
|  | Very much | 27 (14.2) | 18 (13.8) |
| Q22. Have you had a dry cough? | Not at all | 88 (46.3) | 67 (51.5) |
|  | A little | 81 (42.6) | 43 (33.1) |
|  | Quite a bit | 16 (8.4) | 16 (12.3) |
|  | Very much | 5 (2.6) | 4 (3.1) |
| Q23. Have you experienced a decrease in your physical capabilities? | Not at all | 30 (15.8) | 21 (16.2) |
|  | A little | 87 (45.8) | 58 (44.6) |
|  | Quite a bit | 51 (26.%) | 35 (26.9) |
|  | Very much | 22 (11.6) | 16 (12.3) |
| Q24. Has weight loss been a problem for you? | Not at all | 129 (67.9) | 92 (70.8) |
|  | A little | 41 (21.6) | 28 (21.5) |
|  | Quite a bit | 9 (4.7) | 6 (4.6) |
|  | Very much | 11 (5.8) | 4 (3.1) |
| Q25. Have you had pain in the area of surgery? (if you have not had surgery for lung cancer, please select N/A) | Not at all | 35 (18.4) | 23 (17.7) |
|  | A little | 40 (21.1) | 29 (22.3) |
|  | Quite a bit | 20 (10.5) | 7 (5.4) |
|  | Very much | 7 (3.7) | 5 (3.8) |
|  | N/A | 88 (46.3) | 66 (50.8) |
| Q26. Has the area of your wound been oversensitive? (if you have not had surgery for lung cancer, please select N/A) | Not at all | 42 (22.1) | 25 (19.2) |
|  | A little | 35 (18.4) | 26 (20.0) |
|  | Quite a bit | 13 (6.8) | 6 (4.6) |
|  | Very much | 8 (4.2) | 5 (3.8) |
|  | N/A | 92 (48.4) | 68 (52.3) |
| Q27. Have you been restricted in your performance due to the extent of surgery? (if you have not had surgery for lung cancer, please select N/A) | Not at all | 35 (18.4) | 25 (19.2) |
|  | A little | 42 (22.1) | 24 (18.5) |
|  | Quite a bit | 12 (6.3) | 5 (3.8) |
|  | Very much | 8 (4.2) | 6 (4.6) |
|  | N/A | 93 (48.9) | 70 (53.8) |
| Q28. Have you had any difficulty using your arm or shoulder on the side of the chest operation? (if you have not had surgery for lung cancer, please select N/A) | Not at all | 62 (32.6) | 39 (30.0) |
|  | A little | 28 (14.7) | 14 (10.8) |
|  | Quite a bit | 3 (1.6) | 4 (3.1) |
|  | Very much | 4 (2.1) | 3 (2.3) |
|  | N.A | 93 (48.9) | 70 (53.8) |
| Q29. Has your scar pain interfered with your daily activities? (if you have not had surgery for lung cancer, please select N/A) | Not at all | 58 (30.5) | 36 (27.7) |
|  | A little | 29 (15.3) | 16 (12.3) |
|  | Quite a bit | 7 (3.7) | 6 (4.6) |
|  | Very much | 2 (1.1) | 2 (1.5) |
|  | N/A | 94 (49.5) | 70 (53.8) |
